# Evaluation of Community Perceptions and Prevention Practices Related to Ebola Virus as Part of Outbreak Preparedness in Uganda, 2020

**DOI:** 10.9745/GHSP-D-21-00661

**Published:** 2022-06-29

**Authors:** Joseph Musaazi, Apophia Namageyo-Funa, Victoria M. Carter, Rosalind J. Carter, Mohammed Lamorde, Rose Apondi, Tabley Bakyaita, Amy L. Boore, Vance R. Brown, Jaco Homsy, Joanita Kigozi, Aybüke Koyuncu, Maria Sarah Nabaggala, Vivian Nakate, Emmanuel Nkurunziza, Daniel F. Stowell, Richard Walwema, Apollo Olowo, Mohamed F. Jalloh

**Affiliations:** a Infectious Diseases Institute, College of Health Sciences, Makerere University, Kampala, Uganda.; b Center for Global Health, U.S. Centers for Disease Control and Prevention, Atlanta, GA, USA.; c National Center for Emerging and Zoonotic Infectious Diseases, U.S. Centers for Disease Control and Prevention, Atlanta, GA, USA.; d Uganda Ministry of Health, Kampala, Uganda.; e Institute for Global Health Sciences, University of California San Francisco, California, USA.

## Abstract

**Introduction:**

During the 2018–2020 Ebola virus disease (EVD) outbreak in the Democratic Republic of the Congo, risk communication and community engagement (RCCE) were prioritized in geographic areas in Uganda considered at high risk of introduction of EVD. To inform EVD preparedness in Uganda, we evaluated community perceptions and prevention practices related to EVD in 6 districts in Uganda.

**Methods:**

In March 2020, we conducted a population-based survey in 6 purposively selected districts in Uganda using multistage cluster sampling. We examined differences between districts classified as high- versus low risk for EVD in terms of their message exposure from RCCE; risk perception; and EVD knowledge, attitudes, and prevention practices.

**Results:**

A total of 3,485 respondents were interviewed (91% response rate). EVD message exposure was more common in the high- versus low-risk districts. EVD risk perceptions were low overall but greater in the high- versus low-risk districts. Comprehensive knowledge was significantly greater in the high- versus low-risk districts (adjusted prevalence ratio [aPR] 1.61, 95% confidence interval [CI]=1.35, 1.93). Respondents' engagement in all 3 EVD prevention practices (frequent handwashing with soap, avoiding physical contact with suspected Ebola patients, and avoiding burials involving contact with a corpse) was very low (4%). However, respondents with comprehensive knowledge were more likely to engage in all 3 EVD prevention practices compared to respondents without comprehensive knowledge (aPR 1.87, 95% CI=1.08, 3.25).

**Conclusion:**

Our findings suggest that while RCCE efforts as part of EVD outbreak preparedness may have contributed to higher EVD knowledge in the targeted high-risk districts, uptake of prevention behaviors was similarly low across districts. In a non-outbreak setting, implementing targeted RCCE strategies may not be sufficient to motivate people to adopt protective behaviors in the absence of a high threshold of perceived threat such as in an active outbreak.

## Abstract (precis)

Targeted risk communication and community engagement strategies to raise Ebola virus disease awareness and knowledge, particularly in setting where risk of infection is perceived to be low, may not be sufficient to motivate people to adopt protective behaviors and prevention practices.

## Teaser

**Key Findings:**

- Individuals in Ebola virus disease (EVD) high-risk districts reported greater EVD message exposure and comprehensive knowledge compared to individuals in EVD low-risk districts.
- Individuals with comprehensive knowledge of EVD were more likely to self-report frequent handwashing with soap, avoiding physical contact with suspected Ebola patients, and avoiding funerals/burials that involve physical contact with a corpse.
- Rejection of EVD misconceptions was not significantly associated with self-reported EVD-related prevention practices.

**Key Implications:**

- Efforts to promote EVD comprehensive knowledge may facilitate the uptake of EVD prevention practices.
- Risk communication and community engagement efforts continue to be critical for EVD preparedness and response efforts, especially in high-risk geographic areas.

## INTRODUCTION

Ebola virus disease (EVD) is a rare and deadly infectious hemorrhagic fever disease. Four of the 6 known species of ebolavirus can cause disease in humans.^1^ Since 1976, EVD outbreaks have been recorded sporadically in East, Central, and West Africa.^1^^,^^2^ Uganda has experienced 6 documented outbreaks of EVD since 2000 that can be attributed to 3 species of ebolaviruses (Zaire, Sudan, and Bundibugyo).^3^ Despite substantial advances in clinical management, the case fatality ratio (CFR) for EVD remains high, reaching more than 60% for Zaire ebolavirus in 2018–2020.^3^ The public health response to manage EVD outbreaks requires substantial resources for coordination, surveillance, laboratory, case management, infection prevention and control, vaccination, and risk communication and community engagement (RCCE).

Human behavior can influence both the spread and containment of EVD outbreaks. Understanding how community dynamics impact behaviors is important for developing and implementing effective RCCE activities during EVD outbreaks. Assessments of the public's knowledge, attitudes, and practices (KAP) were conducted during the 2014–2016 EVD outbreak in West Africa^4^^–^^7^ and the 2018–2020 outbreak in the Democratic Republic of the Congo (DRC).^8^^,^^9^ These assessments guided the development and implementation of evidence-based RCCE strategies and other interventions to reduce transmission and end the outbreaks.^10^^,^^11^ For instance, KAP studies in West Africa provided information to guide culturally acceptable modifications to care-seeking behaviors and burial rituals involving contact with dead bodies, which were critical to containing the outbreak.^12^^–^^16^ EVD KAP studies have also helped identify misconceptions that have hindered EVD prevention and treatment efforts.^4^^,^^6^^,^^17^ In DRC, KAP surveys and other rapid qualitative assessments similarly informed outbreak response strategies.^18^ During the 2018–2020 outbreak, countries bordering DRC also conducted various community-based assessments to inform context-specific preparedness efforts to prevent and respond to possible importation events.^19^

Understanding how community dynamics impact behaviors is important for developing and implementing effective RCCE activities during EVD outbreaks.

On June 11, 2019, the Uganda Ministry of Health (MOH) declared an EVD outbreak in the Kasese district in the South Western region of Uganda.^20^ The index case and 2 secondary infections were all imported cases linked to the tenth documented EVD outbreak in DRC. The Uganda MOH and response partners promptly responded to and contained this EVD outbreak. As part of its preparedness efforts, Uganda prioritized targeted interventions in 30 districts considered to be at high or moderate risk of introduction of EVD cases from Eastern DRC, where the 2018–2020 DRC EVD outbreak was centered.^21^ High-risk districts were characterized as sharing a border with DRC and having direct access to the affected areas in DRC; moderate-risk districts shared a border with DRC but had no access to the affected areas in DRC. Low-risk districts included the rest of the country except for Kampala and Wakiso, which were designated as high-risk districts. Interventions included community-based surveillance, RCCE using mass media and community-based channels, point-of-entry screenings, infection prevention measures in health facilities, and establishment of holding centers for suspected EVD patients.^21^

We aimed to assess community perceptions and prevention practices related to EVD as part of EVD outbreak preparedness efforts in Uganda during an ongoing outbreak in DRC. To understand the extent of outbreak preparedness RCCE, we explored differences in message exposure and KAP outcomes between districts classified by the Government of Uganda as high versus low risk for EVD, in the context of high-risk districts more heavily targeted with RCCE than the low-risk districts.

## METHODS

### Study Design

In March 2020, we conducted a cross-sectional population-based KAP survey in 4 high-EVD-risk and 2 low-EVD-risk districts in Uganda. We purposively selected the 6 districts using the EVD risk classification of districts developed by the Uganda MOH.^21^ Selection criteria included districts that were considered high risk for EVD importation, close to the Uganda border, or a major business hub in Uganda. The selected high-risk districts included 3 districts that share a border with the DRC nearest to the epicenter of the EVD outbreak (Kasese, Kisoro, and Arua) and the capital, Kampala, which is the major destination of many travelers entering Uganda by any transportation means and the country's busiest business hub. The 2 low-risk districts selected share borders with Kenya and South Sudan (Busia and Lamwo, respectively) and are major business border crossings to and from Uganda. At the Uganda MOH's request, the sample size for this survey was powered to provide district-specific estimates for these 6 districts to inform district-level preparedness efforts. Based on sample size calculations, we estimated that 640 eligible individuals from 20 clusters were required to be approached for consent in each of the 6 districts— for a total target sample of 3,840 participants after adjusting for an expected 25% level of non-responsiveness. Details of the sample size calculations are in the study protocol (Supplement 1).

### Selection of Households and Respondents

Our recruitment targets were household heads (50%), non-household head women aged 25 years and older (25%), and young persons aged 15–24 years (25%). This was to ensure diversity in the sample and include a representative number of women and adolescents/youths. To reach these targets, we randomly selected 20 clusters within districts as defined by the Uganda Bureau of Statistics and enumerated households in the selected clusters with help from local stakeholders including community leaders. Systematic random sampling was used to select up to 16 households per cluster. Households were marked as unreachable after 3 attempts to contact them on separate days in the same week. We did not replace unreachable households because this was accounted for in the sample size estimation. Two eligible individuals were approached in each selected household—first the household head followed by a second randomly selected household member who was either a woman aged 25 years or older or a young person (male or female) aged between 15 and 24 years. We alternated these second household members each time we approached a new household (if available).

### Questionnaire Development and Data Collection

The survey questionnaire was adapted from EVD KAP assessments in Sierra Leone and Guinea (Supplement 2).^4^^,^^6^ The principal investigator led the adaptation of the survey questionnaire and modified some questions to reflect the Ugandan context. The survey questionnaire included questions on respondents' demographic characteristics, EVD awareness and knowledge, risk perception, and self-reported EVD prevention practices. Trained research assistants with previous survey experience in Uganda conducted face-to-face interviews with respondents and collected data electronically using computer tablets programmed with Open Data Kit.^22^ Interviews were conducted in local languages and lasted approximately 1 hour on average. We translated the informed consent and assent forms into local languages. By signing the consent forms, participants were aware that the information collected would be kept confidential and would be used to inform decision making on EVD preparedness.

### Data Analysis

We analyzed the data in Stata version 16.1 Special Edition (StataCorp). We created analytical variables to: (1) enable comparison of data collected from high- versus low-risk districts, (2) account for pre-survey self-reported exposure to EVD prevention messages, and (3) measure respondents' knowledge of all 3 key measures of Ebola prevention and treatment as well as ability to reject 3 key misconceptions about EVD or lack thereof. The following analytical variables were created: (1) a new binary variable to indicate district risk category (i.e., high-districts (coded 1) versus low-risk districts (coded 0)); (2) binary variable to indicate any EVD prevention message exposure (i.e., coded 1 if received any EVD prevention message before the survey and coded 0 otherwise); (3) a composite binary variable to indicate knowledge of all 3 key measures of Ebola prevention and treatment (i.e., coded 1 for knowing that EVD is preventable by avoiding physical contact with infected corpses, early medical care reduces household transmission, and early medical care increases chance of survival; and coded 0 for not knowing all 3 knowledge measures); (4) a binary composite variable to indicate a respondent's ability to reject 3 key misconceptions about EVD (i.e., transmissible by air, spiritual healers can successfully treat EVD, and traditional healers can successfully treat EVD); (5) a binary composite comprehensive knowledge variable was then created to indicate knowledge of all 3 measures of prevention and treatment while also rejecting all 3 EVD misconceptions (coded 1 for having comprehensive knowledge and 0 for not); and (6) a binary composite variable was calculated for self-reporting of all 3 key Ebola prevention practices (i.e., coded 1 for citing more frequent handwashing with soap, avoiding physical contact with suspected Ebola patients, and avoiding funerals/burials that involve physical contact with a corpse; and coded 0 for not citing all these measures). The selected key items were based on the published literature from West Africa showing these 3 items as having performed well in serialized KAP surveys in Sierra Leone in addition to Guinea.^4^^,^^6^

Analyses were performed as frequencies, proportions, and 95% confidence intervals (CI) of proportions comparing high- versus low-risk districts. Results were disaggregated to show district-specific estimates (Supplement 3). We accounted for the survey design at analysis by using inverse probability survey weights. To determine associations, we fitted 3 modified Poisson regression models using generalized estimating equations with exchangeable correlation structure to account for correlation of outcomes due to clustering of respondents.^23^ In the first model, we examined sociodemographic correlates (district risk category, sex, age, education, and religion) of self-reported exposure to any EVD messages in the 6 months before the interview. In the second model, we examined the association between EVD message exposure and comprehensive knowledge, adjusting for demographic covariates previously listed. In the third model, we examined the association between EVD comprehensive knowledge and all 3 prevention practices, adjusting for demographic covariates previously listed. In sensitivity analysis, we modified the third model so that the 2 components of comprehensive knowledge (correct knowledge and rejection of misconceptions) were entered as 2 separate predictor variables. Statistical significance testing was based on a 2-tailed Wald-type test.

We chose a modified Poisson regression approach because the outcomes were common (i.e., >10% prevalence). When the prevalence of the outcome is greater than 10% (as for message exposure), the odds ratio exaggerates the association and is not approximately equal to the prevalence ratio or relative risk; hence we used Poisson regression with robust standard errors to get the adjusted prevalence ratio (aPR) with their 95%CI.^23^ We used variance inflation factors to evaluate multicollinearity in fitted models, wherein variance inflation factors >10 were indicative of severe multicollinearity. We examined the effect of missing data on regression model estimates by refitting models after completing missing data using the multiple imputation chained equation approach.^24^

### Ethical Approval

Ethical approval was obtained from the Uganda Virus Research Institute Research Ethics Committee (UVRI REC GC/127/19/11/756), and the survey was registered with the Uganda National Council for Science and Technology (HS531ES). The Human Subjects Office of the Center for Global Health at the U.S. Centers for Disease Control and Prevention approved the survey as a public health evaluation. All respondents provided individual written or thumb-printed consent (if illiterate) before the interview. For respondents aged 15–17 years, a parent, guardian, or household head provided consent, and the respondent assented. At the end of the interviews in each household, we gave a bar of soap and an EVD education flyer.

## RESULTS

### Respondents' Sociodemographic Characteristics

A total of 3,485 (91%) of the 3,840 individuals approached in the 6 districts consented to participate in the survey: 634 from Busia, 593 from Lamwo, 506 from Arua, 511 from Kampala, 612 from Kisoro, and 629 from Kasese. Across all districts, 51% of the respondents were household heads, 29% were non-household head women aged 25 years and older, and 20% were young people aged 15–24 years. Sixty percent of respondents were female. Sex, educational level, and occupation were statistically significantly different between low-risk and high-risk districts (*P*<.001, =.022, and <.001, respectively) (Table 1).

**TABLE 1.** Characteristics of Knowledge, Attitudes, and Practices Survey Respondents^a^ in 6 Districts, Uganda, 2020

	All Districts, No. (%) (N=3,485)	Low-Risk Districts,^b^ No. (%) (N=1,227)	High-Risk Districts,^c^ No. (%) (N=2,258)	*P* Value^d^
Respondent category				
Household head	1,769 (50.8)	616 (50.2)	1,153 (51.1)	.20
Woman aged ≥25 years	1,019 (29.2)	346 (28.2)	673 (29.8)	
Young person aged 15–24 years	697 (20.0)	265 (21.6)	432 (19.1)	
Age, years^e^				
15–24	761 (21.8)	284 (23.2)	477 (21.1)	.16
25–34	878 (25.2)	286 (23.3)	592 (26.2)	
35–44	715 (20.5)	244 (19.9)	471 (20.9)	
45–59	686 (19.7)	242 (19.7)	444 (19.7)	
60 and above	445 (12.8)	171 (13.9)	274 (12.1)	
Sex				
Female	2,106 (60.4)	693 (56.5)	1,413 (62.6)	<.001
Male	1,379 (39.6)	534 (43.5)	845 (37.4)	
Education level^f^				
No formal education	640 (18.4)	201 (16.4)	439 (19.5)	.02
Primary	1,747 (50.2)	650 (53.1)	1,097 (48.6)	
Secondary and higher	1,093 (31.4)	373 (30.5)	720 (31.9)	
Occupation^f,g^				
Agriculture	1,504 (43.2)	570 (46.6)	934 (41.4)	<.001
Service and sales	572 (16.4)	165 (13.5)	407 (18.0)	
Elementary occupations	253 (7.3)	50 (4.1)	203 (9.0)	
Professional job	201 (5.8)	47 (3.9)	154 (6.8)	
Casual labor	24 (0.7)	6 (0.5)	18 (0.8)	
Unemployed^h^	925 (26.6)	384 (31.4)	541 (24.0)	
Religion^f^				
Christian^i^	3,128 (89.8)	1,109 (90.5)	2,019 (89.5)	.006
Muslim	293 (8.4)	107 (8.7)	186 (8.2)	
Other	62 (1.8)	10 (0.8)	52 (2.3)	

a Response rate: Overall 91%, Low-risk districts 92%, High-risk districts 89%.

b Two low-risk districts: Lamwo and Busia.

c Four high-level districts: Kasese, Kisoro, Arua, and Kampala.

d Pearson Chi-square test P-values for comparing high-risk versus low-risk districts. *P*<.05 is significant and *P*≥0.05 is not significant (at 5% level).

e Age categorized as per the 2016 Uganda Demographic and Health Survey.^25^

f Missing values: Education (n=5, <1%), Occupation (n=6, <1%), Religion (n=2, <1%).

g Occupation categories derived from the 2016 Uganda Demographic and Health Survey.^25^

h Unemployed mostly included students=97 (10.5%).

i Christians include: Anglicans, Catholics, Pentecostals, and Seventh-day Adventists.

### EVD Awareness and Risk Perceptions

Overall, 47% of all respondents were aware of the DRC outbreak at the time of the interview, more frequently so in the high- versus low-risk districts (49% versus 37%; *P*=.001, Table 2). Approximately half of all respondents (52%) perceived themselves to be at some risk of getting EVD; 22% (95% CI=18%, 26%) in the high-risk districts perceived themselves to be at high risk compared to 7% (95% CI=5%, 11%) in the low-risk districts (*P*<.001) (Table 2).

**TABLE 2.** Exposure to EVD Messages and Perceptions of Information Gap in 6 Districts in Uganda, 2020

	All districts, % (95% CI) (N=3,485)	Low-Risk Districts,^a^ % (95% CI) (N=1,227)	High-Risk Districts,^b^ % (95% CI) (N=2,258)	*P* Value^c^
Aware of EVD outbreak in DRC	47.1 (43.2, 51.0)	37.3 (32.0, 42.9)	49.0 (44.5, 53.5)	.001
Perceived risk of contracting EVD^d,e^				
No risk	48.6 (44.2, 52.9)	46.5 (41.4, 51.8)	49.0 (43.9, 54.1)	<.001
Small risk	23.2 (20.9, 25.7)	27.5 (23.3, 32.1)	22.3 (19.7, 25.3)	
Moderate risk	8.9 (7.6, 10.5)	18.9 (14.8, 23.7)	7.0 (5.6, 8.6)	
High risk	19.3 (16.4, 22.6)	7.1 (4.7, 10.5)	21.7 (18.2, 25.7)	
Ebola messages received in past 6 months^d^				
Did not receive any Ebola messages	50.0 (48.0, 51.9)	71.8 (67.0, 76.2)	45.6 (43.5, 47.8)	<.001
Handwashing	34.0 (31.0, 37.2)	15.0 (11.1, 19.9)	37.8 (34.3, 41.4)	<.001
Avoid physical contact with people	31.7 (28.5, 35.1)	14.8 (12.0, 18.2)	35.1 (31.2, 39.1)	<.001
Avoid participating in funeral practices and traditional burials that involve contact with the corpse	18.3 (16.0, 20.8)	7.7 (5.8, 10.1)	20.3 (17.6, 23.4)	<.001
Report sick people to health authorities	15.6 (13.8, 17.6)	8.7 (6.1, 12.1)	16.9 (14.9, 19.2)	<.001
Report deaths that resemble Ebola to health authorities	13.9 (12.2, 15.7)	6.1 (4.3, 8.4)	15.4 (13.4, 17.6)	<.001
Avoid eating bush meat	12.8 (10.4, 15.7)	0.6 (0.3, 1.5)	15.2 (12.3, 18.7)	<.001
Avoid overcrowded places	1.0 (0.6, 1.6)	0.1 (<0.1, 0.4)	1.2 (0.8, 1.9)	<.001
Reported source of EVD information^d,f,g^				
Radio	65.2 (60.8, 69.3)	70.4 (59.7, 79.3)	64.2 (59.3, 68.7)	.28
Television	35.3 (30.8, 40.1)	11.4 (5.8, 21.3)	40.1 (34.8, 45.5)	<.001
Church or mosque	27.5 (20.2,36.2)	6.9 (3.9, 11.9)	31.5 (22.8, 41.8)	<.001
Community meetings	26.3 (20.6, 32.9)	19.6 (14.6, 25.7)	27.6 (20.9, 35.4)	.08
Household visits	20.0 (10.9, 34.0)	7.1 (4.2, 11.7)	22.6 (11.7, 39.2)	.004
Posters or flyers	10.1 (7.1, 14.3)	1.5 (0.6, 3.7)	11.8 (8.2, 16.8)	<.001
Newspapers	8.3 (6.1, 11.3)	4.3 (1.9, 9.4)	9.1 (6.5, 12.6)	.08
Megaphone public announcements	8.0 (3.3, 18.0)	1.7 (0.6, 4.5)	9.2 (3.7, 21.2)	.006
Reported information gap^d,f^				
How EVD is prevented	64.1 (58.6, 69.3)	72.2 (67.3, 76.7)	62.5 (55.9, 68.6)	.02
How EVD is spread	45.8 (40.1, 51.6)	61.0 (54.8, 66.9)	42.8 (36.1, 49.7)	<.001
Signs and symptoms of EVD	40.8 (34.3, 47.7)	59.9 (54.3, 65.2)	37.1 (29.5, 45.3)	<.001
Where to go for EVD treatment	29.8 (24.6, 35.7)	34.2 (26.5, 42.9)	28.9 (22.9, 35.8)	.31
How to care for an EVD patient	20.1 (15.1, 26.2)	29.3 (23.0, 36.5)	18.3 (12.6,25.7)	0.026

Abbreviations: CI, confidence interval; DRC, Democratic Republic of the Congo; EVD, Ebola virus disease.

a Two low-risk districts: Lamwo and Busia.

b Four high-level districts: Kasese, Kisoro, Arua, and Kampala.

c Design-based F-statistic p values for comparing high-risk versus low-risk districts. *P*<.05 is significant and *P*≥.05 is not significant (at 5% level).

d Missing values: Perceived risk of contracting EVD (n=51, 1%), preventive Ebola messages received (n=113, 3%), source of EVD information (n=44, 2%), EVD information gap (n=2,130, 61%).

e Don't know responses excluded from the risk perception item (n=828, 24%).

f Multiple response questions: Only the key items presented, detailed responses are presented in Supplement 2.

g Denominator was number of participants who reported ever received preventive EVD messages.

### EVD Message Exposure, Sources of Information, and Information Gaps

Half of all respondents were never exposed to EVD messages in the 6 months before the interview, which was more common in the low-risk districts (72%) compared to the high-risk districts (46% *P*<.001, Table 2). EVD prevention messages were more common in the high- versus low-risk districts for the following: handwashing (38% versus 15%, *P*<.001); avoiding physical contact with people (35% versus 15%, *P*<.001); avoiding unsafe burials (20% versus 8%, *P*<.001). Radio was the most common source of EVD information in both low- and high-risk districts (65%) followed by television (35%) and churches or mosques (27%) and by community meetings (26%). Respondents from low-risk compared to districts were more likely to want more information about EVD prevention (72% versus 63%, *P*=.02), transmission (61% versus 43%, *P*<.001), and signs and symptoms (60% versus 37%, *P*<.001) (Table 2).

### EVD Knowledge and Prevention Practices

Overall, 5% of all respondents were able to mention a total of 4 key signs/symptoms of EVD (fever, severe headache, diarrhea, and vomiting) without any prompting by the interviewer (Table 3). Fever was more frequently mentioned as an EVD symptom in high- versus low-risk districts (46% versus 31%; *P*=.001). Overall, 63% of respondents had knowledge of the 3 key measures of EVD prevention and treatment, and such knowledge was greater in the high- versus low-risk districts (65% versus 55%, *P*=.002). The ability to reject the 3 measures of EVD misconceptions was more common in the high- versus low-risk districts (73% versus 57%, *P*<.001). Taken together, comprehensive knowledge was greater in the high- versus low-risk districts (48% versus 32%, *P*<.001) (Table 3).

**TABLE 3.** Ebola-Related Knowledge, Attitudes, Intentions, and Self-Reported Behaviors in 6 Districts in Uganda, 2020

	All Districts, % (95% CI) (N=3,485)	Low-Risk Districts, % (95% CI) (N=1,227)	High-Risk Districts, % (95% CI) (N=2,258)	*P* Value^a^
Knowledge of EVD signs/symptoms^b,c^				
Vomiting	51.0 (47.9, 54.1)	57.6 (52.2, 62.8)	49.7 (46.1, 53.3)	.02
Diarrhea	50.4 (45.0, 55.7)	50.3 (44.7, 55.9)	50.4 (44.1, 56.7)	.99
Fever	43.6 (40.3, 46.9)	30.5 (25.7, 35.7)	46.1 (42.3, 50.0)	.001
Severe headache	21.5 (19.5, 23.6)	22.9 (18.8, 27.7)	21.2 (19.0, 23.6)	.50
Knowledge of all 4 above signs/symptoms	4.8 (3.4, 6.7)	5.0 (3.1, 7.8)	4.7 (3.2, 7.1)	.87
Knowledge of EVD prevention and treatment				
Early medical care increases chance of survival^b^	85.4 (83.3, 87.2)	80.2 (77.2, 82.9)	86.4 (84.0, 88.5)	.001
Early medical care reduces household transmission^b^	82.2 (79.3, 84.9)	78.5 (75.2, 81.6)	83.0 (79.4, 86.0)	.06
EVD prevention by avoiding contact with infected corpses^b^	76.8 (74.0, 79.4)	68.7 (63.1, 73.8)	78.4 (75.2, 81.3)	.001
Knowledge of all 3 EVD knowledge measures^b^	63.0 (60.1, 65.8)	54.7 (49.2, 60.1)	64.7 (61.4, 67.8)	.002
Misconceptions of EVD				
EVD is transmissible by air^b^	22.2 (19.6, 25.0)	42.6 (35.8, 49.8)	18.1 (15.4, 21.2)	<.001
Spiritual healers can successfully treat EVD^b^	9.7 (7.9, 11.8)	7.7 (5.5, 10.7)	10.1 (8.0, 12.6)	.18
Traditional healers can successfully treat EVD^b^	2.9 (2.3, 3.7)	4.7 (3.0, 7.5)	2.6 (2.0, 3.4)	.03
Rejection of all 3 EVD misconception measures	70.4 (67.6, 73.0)	56.6 (51.0, 62.0)	73.1 (69.9, 76.0)	<.001
Comprehensive knowledge^d^	45.4 (41.8, 49.2)	32.1 (26.2, 38.7)	48.1 (43.8, 52.3)	.001
Attitudes toward EVD survivors				
Would welcome back survivor into the community^b^	61.8 (58.4, 65.2)	57.3 (52.2, 62.3)	62.8 (58.8, 66.6)	.09
Ebola survivor student does not put class at risk of EVD^b^	56.4 (52.0, 60.6)	46.8 (42.5, 51.3)	58.3 (53.1, 63.2)	.001
Would buy fresh vegetables from survivor shopkeeper^b^	47.3 (43.8, 50.9)	50.0 (45.2, 54.9)	46.8 (42.6, 51.0)	.31
Attitudes toward safe burial practices				
Accept safe alternatives to traditional burial rituals	80.6 (77.1, 83.6)	82.9 (78.2, 86.7)	80.1 (76.1, 83.6)	.34
Intention if family member is suspected of EVD^b,c^				
Take the family member to a health facility	52.4 (46.3, 58.5)	64.5 (58.2, 70.3)	50.0 (42.9, 57.2)	.003
Report to district health authorities	43.3 (38.8, 48.0)	39.2 (35.3, 43.3)	44.2 (38.8, 49.6)	.15
Avoid all physical contact	10.3 (7.8, 13.6)	8.0 (5.9, 10.8)	10.8 (7.8, 14.8)	.18
Do nothing	5.2 (3.8, 6.9)	9.6 (6.9, 13.2)	4.3 (2.9, 6.4)	.002
Help care for the family member at home	2.6 (1.7,4.0)	5.1 (3.4, 7.6)	2.1 (1.1, 3.9)	.02
Hide the EVD suspected family member	<0.1 (<0.1, 0.1)	0.1 (<0.1, 0.5)	<0.1 (<0.1, 0.1)	.07
Self-reported participation in recent funeral or burial				
Self-reported participation in a funeral/burial in past month	39.3 (35.7, 43.0)	41.3 (35.7, 47.1)	38.9 (34.8, 43.2)	.51
Religious leader prayed for the deceased^c,e^	84.9 (78.7, 89.5)	90.5 (86.7, 93.4)	83.8 (76.3, 89.2)	.05
Family members observed burial from a distance^c,e^	6.2 (3.8, 9.9)	10.0 (6.0, 16.2)	5.4 (2.9, 10.0	.13
Attendees performed rituals involving contact with corpse^d,f^	25.5 (17.4, 35.8)	18.5 (13.0, 25.7)	26.9 (17.3, 39.1)	.18
Attendees touched each other (e.g., hugs and handshakes)^c,e^	50.8 (42.7, 58.8)	61.2 (54.3, 67.7)	48.7 (39.2, 58.3)	.04
Respondent had physical contact with the corpse^e^	11.1 (8.4, 14.7)	10.1 (7.2, 14.0)	11.3 (8.1, 15.6)	.63
Self-reported EVD prevention practices^c,f^				
Self-reported any Ebola prevention practice	67.3 (63.3, 71.1)	72.4 (64.3, 79.2)	66.3 (61.7, 70.6)	.18
Wash hands with soap and water more frequently	57.1 (52.0, 62.0)	57.2 (46.1, 67.6)	57.0 (51.3, 62.5)	.98
Avoid physical contact with suspected Ebola patients	22.9 (19.3, 26.8)	26.7 (20.0, 34.7)	22.1 (18.2, 26.6)	.28
Avoid burials that involve contact with a corpse	13.3 (10.1, 17.2)	12.6 (8.7, 17.0)	13.4 (9.8, 18.1)	.80
Reported all 3 prevention practices	4.0 (2.0, 7.8)	2.5 (1.1, 6.0)	4.3 (2.0, 9.0)	.37

Abbreviation: CI, confidence interval; EVD, Ebola virus disease.

a Design-based F-statistic *P* values; comparing high-risk versus low-risk districts. *P*<.05 is significant and *P*≥.05 is not significant (at 5% level).

b Missing values: Knowledge of EVD prevention and treatment (n=66, 2%); knowledge of EVD signs/symptoms (n=792, 23%); traditional healers can successfully treat EVD (n=66, 2%); spiritual healers can successfully treat EVD (n=66, 2%); EVD is transmissible by air (n=518, 15%); comprehensive knowledge (n=523, 15%). Would welcome back EVD survivor into the community (n=79, 2%); would buy fresh vegetables from EVD survivor. shopkeeper (n=91, 3%), Ebola survivor student does not put class at risk of EVD (n=92, 3%); would avoid touching or washing the corpse of a family member (n=48, 1%); intended practice if family member is suspected of EVD (n=627, 18%); participated in a funeral/burial in past month (n=30, <1%); features of the funeral/burial ceremony (n=64, 4%); respondents had physical contact with corpse at the funeral/burial (n=33, 2%).

c Multiple response questions: Only the key items presented here. Detailed responses are presented in Supplement 2.

d Comprehensive knowledge is defined as composite of knowledge of all 3 EVD knowledge measures and rejection of all 3 EVD misconception measures.

e Denominator is number of respondents who reported to have participated in a funeral/burial in past month.

f Denominator is number of respondents who reported that they took action to avoid getting EVD.

### Attitudes and Behavioral Intentions Toward Care and Burial

If a family member was suspected to have EVD, 52% of all respondents intended to take the person to a health facility (50% in high-risk districts and 65% in low-risk districts, *P*=.003; Table 3) while 43% of all respondents intended to report the suspected case to the health authorities. Overall, 3% of respondents mentioned an intention to provide care at home, and <0.1% mentioned an intention to hide the EVD suspected family member. In a hypothetical scenario, most respondents (62%) said they would welcome an EVD survivor back into the community.

Intention to accept safe alternatives to traditional burials if a family member died of EVD was high overall (81%) (Table 3). Less than half of all respondents (39%) reported having attended a funeral/burial within the past month of the interview (for a death of any cause, not specific to EVD) (Table 3). Among those who attended a recent funeral/burial, 11% reported they directly had some physical contact with the corpse and 51% said that they observed attendees touching each other (e.g., hugging and shaking hands). Respondents in the low-risk districts were more likely to report that attendees touched each other (61%) compared to those in the high-risk districts (49%, *P*=.036) (Table 3).

### Self-Reported Prevention Practices

Self-reported uptake of any EVD prevention practice was 67% overall, without a significant difference between high- versus low-risk districts (66% versus 72%, *P*=.180, Table 3). Among those who reported any uptake, 57% mentioned handwashing frequently with soap and water, 23% mentioned avoiding physical contact with suspected EVD patients, and 13% mentioned avoiding burials involving contact with a corpse. Uptake of all 3 of these prevention practices was very low in both high- and low-risk districts (3% versus 4%, respectively) (Table 3).

### Multivariable Models for EVD Message Exposure, Comprehensive Knowledge, and Uptake of Prevention Practices

#### Association Between Sociodemographic Variables and EVD Message Exposure

EVD message exposure was significantly greater in the high-risk districts (aPR: 2.46; 95% CI=2.09, 2.91) and among male respondents (aPR: 1.09; 95% CI=1.03, 1.14) but less prevalent among respondents with no formal education (aPR: 0.86; 95% CI=0.78, 0.94), adjusting for district category, sex, age, educational level and religion (Table 4).

**TABLE 4.** Sociodemographic Correlates of EVD Message Exposure in 6 Districts in Uganda, 2020

	Total N=3,397^a^	Prevalence of EVD Messages Exposure^b^	Adjusted Model for EVD Messages Exposure^c^	
		% (95% CI)	aPR (95% CI)	*P* Value^d^
District category				
Low risk	1,168	27.4 (23.0, 32.3)	Reference	
High risk	2,229	50.9 (43.3, 58.4)	2.46 (2.09, 2.91)	<.001^e^
Sex				
Female	2,054	46.2 (41.3, 51.2)	Reference	
Male	1,343	56.4 (45.5, 66.7)	1.09 (1.03, 1.14)	.002^e^
Age, years^f^				
15–24	732	57.6 (53.2, 61.8)	Reference	
25–34	863	38.8 (25.2, 54.4)	1.04 (0.96, 1.12)	.39
35–44	698	46.9 (40.6, 53.2)	0.99 (0.93, 1.07)	.95
45–59	669	59.8 (50.0, 68.8)	1.06 (0.98, 1.14)	.14
60 and older	435	50.8 (40.7, 60.9)	0.96 (0.87, 1.05)	.36
Educational level				
No formal education	629	58.4 (49.2, 67.0)	0.86 (0.78, 0.94)	.001^e^
Primary	1,700	50.7 (43.8, 57.5)	0.97 (0.91, 1.03)	.27
Secondary or higher	1,068	45.5 (39.1, 52.2)	Reference	
Religion				
Christian	3,052	51.6 (43.3, 59.7)	Reference	
Muslim	283	38.1 (28.0, 49.3)	1.04 (0.93, 1.17)	.48
Other	62	30.0 (16.4, 48.4)	0.97 (0.79, 1.20)	.79

Abbreviations: aPR, adjusted Prevalence Ratio estimated using Generalized Estimating Equation (GEE) modified Poisson model; CI, confidence interval; EVD, Ebola virus disease.

a Analysis was performed on complete cases (N=3,397) on all factors listed in the table. 529/3,485 (15.2%) respondents had missing data: on EVD message exposure (n=81), educational level (n=5), religion (n=2).

b Percent of total respondents in a specific category (weighted row percentages).

c Estimates from multivariable GEE modified Poisson model including a priori selected factors as presented in the table. There was no evidence of multicollinearity among predictors included in multivariable model; variance inflation factor for all factors included in multivariable model was <3.

d *P*<.05 is significant and *P*≥.05 is not significant (at 5% level).

e Adjusted model results after completing missing values using multiple imputation using chained equations (MICE): N=3,485, District type (high-risk versus low-risk: aPR: 2.45, 95% CI=2.08, 2.88; P<.001), gender (male versus female: aPR:1.09, 95% CI=1.04, 1.15; *P*=.001), and educational level (reference=Secondary+ versus No formal education aPR: 0.85, 95% CI=0.77, 0.94; *P*=.001, Primary aPR: 0.97, 95%CI=0.91, 1.03; *P*=.315).

f Age categorized as per the 2016 Uganda Demographic and Health Survey.^25^

#### Association Between EVD Message Exposure and Comprehensive Knowledge

There was no sufficient evidence of association between EVD message exposure and comprehensive knowledge at complete case analyses (aPR: 1.11; 95% CI=0.99, 1.24, Table 5), but the association was significantly positive after completing missing values using multiple imputation (aPR: 1.12, 95% CI=1.01, 1.24). Comprehensive knowledge was significantly greater in the high-risk districts (aPR: 1.61; 95% CI=1.35, 1.93), and among male respondents (aPR: 1.18; 95% CI=1.08, 1.28), but less prevalent among respondents with no formal education (aPR: 0.87; 95% CI=0.76, 0.98), adjusting for EVD message exposure, district category, sex, age, educational level, and religion (Table 5).

**TABLE 5.** Association Between EVD Message Exposure and Comprehensive Knowledge, Adjusted for Sociodemographic Characteristics - Uganda, 2020

	Total N=2,934^a^	Comprehensive Knowledge^b^ % (95% CI)	Adjusted Model for Comprehensive Knowledge^c^	
			aPR (95% CI)	*P* Value^d^
Received any EVD message				
No	1,120	42.6 (36.8, 48.6)	Reference	
Yes	1,814	49.6 (44.4, 54.8)	1.11 (0.99, 1.24)	.05^e^
District category				
Low-risk	940	31.7 (25.4, 38.7)	Reference	
High-risk	1,994	47.4 (42.2, 52.6)	1.61 (1.35, 1.93)	<.001^e^
Sex				
Female	1,746	42.1 (34.6, 50.0)	Reference	
Male	1,188	55.6 (46.3, 64.5)	1.18 (1.08, 1.28)	<.001^e^
Age (years)^f^				
15–24	639	47.8 (35.8, 60.0)	Reference	
25–34	769	43.7 (35.9, 51.9)	1.05 (0.94, 1.18)	.39
35–44	612	58.4 (47.4, 68.7)	1.13 (1.02, 1.26)	.032
45–59	584	39.8 (24.3, 57.6)	1.10 (0.97, 1.25)	.13
60 and older	330	35.2 (24.2, 47.9)	0.95 (0.81, 1.11)	.52
Educational level				
No formal education	489	41.5 (34.1, 49.3)	0.87 (0.76, 0.98)	.027
Primary	1,474	37.9 (26.2, 51.2)	0.92 (0.84, 1.00)	.05^e^
Secondary or higher	971	55.4 (48.0, 62.5)	Reference^e^	
Religion				
Christian	2,636	45.8 (41.5, 50.1)	Reference	
Muslim	244	50.5 (39.3, 61.6)	1.01 (0.87, 1.17)	.92
Other	54	39.1 (28.9, 50.2)	1.06 (0.79, 1.41)	.71

Abbreviations: aPR, adjusted Prevalence Ratio estimated using Generalized Estimating Equation (GEE) modified Poisson model; CI, confidence interval; EVD, Ebola virus disease.

a Analysis was performed on complete cases (N=2,934) on all factors listed in the table. 551/3,485 (15.8%) respondents had missing data: on any component of comprehensive knowledge 523/3485 (15%), educational level (n=5), religion (n=2).

b Percent of total respondents in a specific category (weighted row percentages).

c Estimates from multivariable GEE modified Poisson model including priori selected factors as presented in the table. There was no evidence of multicollinearity among predictors included in multivariable model; variance inflation factor [VIF] for all factors included in multivariable model was <3.

d *P*<.05 is significant and *P*≥.05 is not significant (at 5% level).

e Adjusted model results after completing missing values using multiple imputation using chained equations (MICE): N=3,485, EVD message exposure (aPR: 1.12, 95% CI=1.01, 1.24; P=.032), district type (high risk versus low risk: aPR=1.64, 95% CI=1.41, 1.92; *P*<.001), gender (male versus female: aPR:1.18, 95% CI=1.08, 1.28, *P*<.001), and Educational level (reference=Secondary+ versus No formal education aPR: 0.87, 95% CI=0.76, 0.99, *P*=.036, primary aPR: 0.92, 95% CI=0.84, 1.00; *P*=.051).

f Age categorized as per the 2016 Uganda Demographic and Health Survey.^25^

#### Association Between EVD Comprehensive Knowledge and Uptake of Prevention Practices

Comprehensive knowledge was positively significantly associated with self-reporting of the 3 EVD prevention practices (aPR: 1.87; 95% CI=1.08, 3.25, Table 6), adjusting for district category, sex, age, educational level, and religion. None of the demographic variables were significantly associated with uptake of the 3 prevention practices. In sensitivity analyses when we examined the 2 domains of comprehensive knowledge as separate covariates in the model, knowing the 3 measures of EVD prevention and treatment (domain 1) was significantly associated with uptake of the 3 prevention practices (aPR: 2.16; 95% CI=1.08, 4.32, Table 6); however, the ability to reject all 3 EVD misconceptions (domain 2) was not significantly associated with the self-reported prevention practices. Multicollinearity was not observed in any of the multivariable models fitted.

**TABLE 6.** Association Between EVD Comprehensive Knowledge and Uptake of Prevention Practices, Adjusted for Sociodemographic Characteristics in 6 Districts in Uganda, 2020

	Total N=1,629^a^	Prevention Practices^b^ % (95% CI)	Adjusted Model for Prevention Practices^c^	
			aPR (95% CI)	*P* Value
Comprehensive knowledge				
No	805	4.0 (2.2, 8.4)	Reference	
Yes	824	4.8 (2.2, 10.3)	1.87 (1.08, 3.25)	.026^d^
District category				
Low-risk	260	2.5 (0.9, 6.5)	Reference	
High-risk	1,369	4.7 (2.4, 8.9)	1.25 (0.55, 2.83)	.59^d^
Sex				
Female	971	3.8 (2.0, 7.0)	Reference	
Male	658	6.2 (2.6, 14.0	1.50 (0.84, 2.67)	.17^d^
Age, years^e^				
15–24	365	5.1 (2.0, 12.4)	Reference	
25–34	424	4.4 (1.8, 10.2)	1.28 (0.68, 2.42)	.45
35–44	346	6.7 (3.0, 14.1)	1.17 (0.57, 2.40)	.67
45–59	319	3.0 (1.1, 8.4)	0.62 (0.25, 1.51)	.29
60 and older	175	2.2 (0.3, 13.7)	0.31 (0.08, 1.23)	.10
Educational level				
No formal education	276	11.8 (5.1, 25.1)	1.49 (0.67, 3.33)	.33
Primary	797	3.1 (1.2, 7.3)	0.67 (0.42, 1.09)	.11
Secondary or higher	556	4.2 (1.5, 11.0)	Reference	
Religion				
Christian	1,451	4.5 (2.4, 8.2)	Reference	
Muslim	140	5.9 (1.8, 17.7)	0.97 (0.39, 2.41)	.95
Other	38	0.5 (0.1, 3.5)	0.71 (0.13, 3.79)	.69

Abbreviations: aPR, adjusted Prevalence Ratio estimated using Generalized Estimating Equation (GEE) modified Poisson model; CI, confidence interval; EVD, Ebola virus disease.

a Analysis was performed for respondents who reported to ever heard of EVD and took action to avoid getting EVD (n=1,718) and had complete cases (N=1,629) on all factors listed in the table. 89/1,718 (5%) respondents had missing data: on comprehensive knowledge variable (n=87), educational level (n=2), religion (n=1).

b Percent of total respondents in a specific category (row percentage).

c Estimates from multivariable GEE modified Poisson model including priori selected factors as presented in the table. There was no evidence of multicollinearity among predictors included in multivariable model; variance inflation factor for all factors included in multivariable model was <3. In separate models for “Knowledge of all 3 key knowledge measures”: (aPR:2.16, 95% CI=1.08, 4.32; *P*=.029), and “Rejected all 3 key EVD misconceptions”: aPR:1.39 (95% CI=0.68, 2.85; *P*=.361).

d Adjusted model results after completing missing values using multiple imputation using chained equations (MICE): N=3,485, comprehensive knowledge (aPR: 1.84, 95% CI=1.06, 3.21; *P*=.031), District type (high risk versus low risk: aPR: 1.04, 95%CI=0.44, 2.47; *P*=.927), gender (male versus female: aPR: 1.52, 95% CI=0.85, 2.72; *P*=160).

e Age categorized as per the 2016 Uganda Demographic and Health Survey.^25^

## DISCUSSION

Our population-based evaluation revealed our empirical understanding of the differences between the high- versus low-risk districts in Uganda in terms of EVD perceptions and prevention practices. Overall, although EVD exposure and EVD comprehensive knowledge were greater in the high- versus low-risk districts, uptake of prevention practices was low across all districts. Adjusted analysis suggested that comprehensive knowledge was associated with EVD message exposure and EVD prevention practices. Deeper analysis of comprehensive knowledge revealed that while knowledge of EVD prevention and treatment was associated with EVD prevention practices, this was not the case with the respondents' rejection of EVD misconceptions. Our findings may reflect the targeted approach implemented by the Government of Uganda to prioritize preparedness efforts in the high-risk districts.^21^

Deeper analysis of comprehensive knowledge revealed that while knowledge of EVD prevention and treatment was associated with EVD prevention practices, this was not the case with respondent rejection of EVD misconceptions.

To our knowledge, this is the first population-based evaluation in Uganda to examine differences between high- versus low-risk districts after a year of intensified EVD preparedness efforts including RCCE interventions. Given the ongoing threat of EVD outbreaks in Uganda, our findings could serve as a baseline for future evaluations of the population-level reach and impact of RCCE interventions for EVD prevention in Uganda. The findings also shed light on areas for additional RCCE activities for consideration. In low-risk districts, for instance, we found that respondents wanted information about EVD prevention, transmission, signs/symptoms, and how to care for an EVD patient, possibly reflecting the RCCE focus placed on high-risk districts. Respondents from high-risk areas were more likely to mention fever as an EVD symptom, which may have been reinforced by body temperature screenings in these areas.^21^ Overall, less than 1 in every 10 respondents knew 4 of the signs and symptoms of EVD (fever, severe headache, diarrhea, and vomiting). Greater investments in the high-risk compared to the low-risk districts explain the greater EVD exposure and EVD comprehensive knowledge among respondents in the high- versus low-risk districts. Having a greater sense of personal risk and being in closer proximity to the outbreak offers another explanation. The reported low EVD message exposure in Kampala, a high-risk district, was noteworthy given it was heavily targeted with intensified RCCE efforts.

Mistrust in authorities and the health system has been shown to influence care-seeking behaviors and uptake of promoted practices.^25^^–^^27^ For instance, in past EVD outbreaks in West Africa and DRC, there were instances when family members hid their loved ones at home instead of reporting them to health authorities and secretly buried them instead of requesting a safe and dignified burial by specially trained teams. In our evaluation, respondents expressed willingness to accept safe and dignified burial measures if a family member died at home and intended to report an EVD suspected family member to the health authorities or take them to a health facility. However, it should be noted that in Sierra Leone, for example, although a similar assessment showed low intention to hide EVD suspected family members^5^ in practice, family members continued to hide sick family members and many times cared for them at home instead of reporting to health authorities. Caring for a sick family member at home is not necessarily synonymous with hiding them from authorities. There are situations where families may report suspected cases to the authorities but are unable to get a timely ambulance service.^28^ While waiting for medical help to arrive, families may feel compelled to provide care for their loved ones,^29^ which shows a need for RCCE efforts to educate on how to safely provide care as they wait for help to come. During EVD outbreaks, it is not only important to strengthen public confidence in the outbreak response services but also equally important to ensure that the promoted services can meet the public's demand and expectations for quality—including showing respect to communities, families, and their loved ones.

Although respondents were exposed to EVD messages and had EVD knowledge, many did not engage in EVD prevention practices. During all EVD outbreaks in sub-Saharan Africa, traditional burial practices involving contact with corpses have been a major source of virus transmission.^13^^,^^16^ In the 2018–2020 DRC outbreak, people living in unaffected areas in DRC struggled to make the connection between the spread of EVD and traditional funeral practices.^30^ Our findings showed that although Uganda was on high alert for EVD, traditional practices for funerals and burials continued both in the high- and low-risk districts. Most respondents intended to accept safe alternatives to traditional funerals and burials if a family member died of EVD. We found that nearly 4 in every 10 respondents had participated in a traditional funeral or burial in the past month. One in every 10 respondents reported their own direct contact with the corpse, which was a more common practice in the low-risk districts. This finding suggests that although respondents were knowledgeable, they may have felt confident that the deaths were not EVD. A possible reason for this confidence is the low number of EVD-related deaths in Uganda at the time.^3^ As was demonstrated during the 2014–2016 EVD outbreak in Sierra Leone, RCCE can be helpful in socializing and explaining the need for safe burials when guidance changes.^13^ Another possibility is that transferring knowledge alone is not sufficient if not supported by actual engagement and behavioral drill practices by the target community, as has been well documented.^31^

Although respondents were exposed to EVD messages and had EVD knowledge, many did not engage in prevention practices.

Misconceptions about EVD in communities can play a role in individuals' understanding of the transmission, prevention, and treatment of EVD. Studies on misconceptions about EVD have found beliefs such as spiritual prayer protects one from EVD and traditional healers treat EVD better than medical doctors.^17^ National EVD KAP surveys in Sierra Leone (2014) and Guinea (2015) found that participants held misconceptions about EVD transmission such as EVD can be transmitted by mosquitoes.^4^^,^^6^ We found that the ability to reject the 3 measures of EVD misconceptions (i.e., transmissible by air, curable by spiritual healers, and curable by traditional healers) was more common in the high- versus low-risk districts. We also found that the rejection of EVD misconceptions was not significantly associated with the uptake of EVD prevention practices. Together with our findings on burial practices, this points to the fact that science-based knowledge could coexist with religious and traditional beliefs^32^ and that misconceptions do not necessarily translate into lower uptake of protective behaviors. Our findings suggest more emphasis should be placed on integrating actionable knowledge and addressing misconceptions through community engagement efforts that can translate into safe and effective prevention and care practices in both high- and low-risk contexts.^33^

The Uganda MOH's RCCE efforts used television, radio, newspapers, posters, and door-to-door health education as part of EVD preparedness.^21^ While mass media may help to quickly raise awareness and create knowledge about EVD before or during the early part of an outbreak,^4^ improvements in attitudinal and behavioral outcomes take much longer and require more targeted comprehensive community engagement.^11^

### Limitations

Our evaluation has several limitations. Due to the cross-sectional design, we cannot establish any causal relationships in the associations that we documented in our results. The binary variables used in the analysis may not capture the latent processes driving the perceptions and practices of the respondents. In addition, respondents may have provided socially desirable responses, especially due to their possible awareness of public health messages. Social desirability may have been more pronounced in the high-risk districts where EVD messages were widely disseminated which could have resulted in an overestimation of the association between district risk level and self-reported prevention practices. Some practical aspects of the survey's administration posed limitations. For instance, because the survey was conducted during working hours, some sampled households in Kampala did not have any eligible household members available and had to be skipped. Furthermore, female respondents were over-represented in our sample compared to the Uganda population possibly because more females were available at the time of our visit or more males declined to be interviewed, which we are unable to discern. Lastly, our results may not be generalizable to all districts categorized as high- versus low-risk for EVD in Uganda because we only evaluated 6 districts. However, a strength of our evaluation is that we have generalizable results for each of the 6 districts to inform future targeted EVD preparedness at the district level.

## CONCLUSION

EVD comprehensive knowledge and message exposure were more common in high- versus low-risk districts following RCCE efforts as part of EVD outbreak preparedness in Uganda. The RCCE efforts in Uganda included extensive use of the media and door-to-door education to share information on EVD signs and symptoms, seeking care, and safe burials. Our findings suggest that while RCCE efforts may have contributed to higher knowledge in the targeted high-risk districts, uptake of prevention behaviors was similarly low across districts. In a non-outbreak setting, implementing targeted RCCE strategies may not be sufficient to motivate people to adopt protective behaviors in the absence of a perceived threat such as in an active outbreak. Additional strategies may be required for comprehensive community engagement in prevention practices.

## Supplementary Material

GHSP-D-21-00661-supplement-2.pdf

GHSP-D-21-00661-supplement-1.pdf

GHSP-D-21-00661-supplement-3.pdf

## Notes

**Peer Reviewed**

**First published online:** June 6, 2022.
