# Supplementary material for: Evaluation of Community Perceptions and Prevention Practices Related to Ebola Virus as Part of Outbreak Preparedness in Uganda, 2020
Source: Glob Health Sci Pract. 2022 Jun 29;10(3):e2100661. doi: 10.9745/GHSP-D-21-00661 (PMC9242603; doi:10.9745/GHSP-D-21-00661)
Supplement: GHSP-D-21-00661-supplement-2.pdf [file GHSP-D-21-00661-supplement-2.pdf]

Supplement to: Musaazi J, Namageyo-Funa A, Carter VM, et al. Evaluation of community perceptions and prevention practices related to Ebola virus as part of outbreak preparedness in Uganda, 2020. *Glob Health Sci Pract*. 2022;10(3):e2100661. https://doi.org/10.9745/GHSP-D-21-00661

Supplement 2: Questionnaire

Assessment of Public Knowledge, Attitudes, and Practices Relating to Ebola Virus Disease Prevention and Treatment in Uganda, 2019

Data Collection Team Identification

Team ID:	Enumerator ID:	Supervisor ID:
Date:	Time:	Location:

District, Cluster, and Household Identification

**DISTRICT**<br>1. Kasese<br>2. Kisoro<br>3. Arua<br>4. Greater Kampala<br>5. Lamwo<br>6. Busia<br><br>**CLUSTER**<br>Identification number ____ ____ ____<br>Name: ______________________________	**HOUSEHOLD**<br>Identification number ____ ____ ____<br>Household size:____ ____<br>Respondent category:<br>____ Head of household<br>____ Woman 25 years and above<br>____ Young person ages 15-24 years

***Indicates core items adapted from EVD KAP assessments from West Africa*

**Indicates new items developed specifically for the Uganda EVD preparedness context*

Supplement to: Musaazi J, Namageyo-Funa A, Carter VM, et al. Evaluation of community perceptions and prevention practices related to Ebola virus as part of outbreak preparedness in Uganda, 2020. *Glob Health Sci Pract*. 2022;10(3):e2100661. https://doi.org/10.9745/GHSP-D-21-00661

SOCIODEMOGRAPHIC CHARACTERISTICS OF RESPONDENT

1. **Gender**
   a. Male
   b. Female

2. **Age:** ___________ years
   or Year of birth: _________
   (enter 88 if don't know and 99 if declined to respond)

3. **What is your highest level of education attained?** *(single selection; prompted as needed)*
   (any level attended even if not completed)
   a. No formal education
   b. Primary school
   c. Secondary school
   d. Post-secondary and above
   e. Declined to respond

4. **What kind of work do you currently do?** *(single selection; prompted as needed)*
   a. Professional / managerial / technical / assistant professional
   b. Clerical support
   c. Service and sales
   d. Skilled agriculture / forestry / fishery
   e. Craft and related trade
   f. Plant and machine operator / assembler
   g. Elementary occupations
   h. Unemployed
   i. Other (specify)
   j. Declined to respond

5. **What is your religion?** *(single selection; unprompted)*
   a. Catholic
   b. Anglican
   c. Muslim
   d. Pentecostal
   e. Seventh Day Adventist
   f. Other (specify)
   g. Declined to respond

Supplement to: Musaazi J, Namageyo-Funa A, Carter VM, et al. Evaluation of community perceptions and prevention practices related to Ebola virus as part of outbreak preparedness in Uganda, 2020. *Glob Health Sci Pract*. 2022;10(3):e2100661. https://doi.org/10.9745/GHSP-D-21-00661

GENERAL HEALTH

[I will now start by asking you to tell me your opinion about health services in your district]

6. **How confident are you in the health services in your district to treat malaria?****
   (single selection; prompted)
   a. Not at all confident
   b. Somewhat confident
   c. Very confident
   d. Never heard of malaria
   e. Declined to respond

7. **How confident are you in the health services in your district to treat tuberculosis (TB/dry cough)?****
   (single selection; prompted)
   a. Not at all confident
   b. Somewhat confident
   c. Very confident
   d. Never heard of tuberculosis
   e. Declined to respond

8. **How confident are you in the health services in your district to treat Ebola?****
   (single selection; prompted)
   a. Not at all confident
   b. Somewhat confident
   c. Very confident
   d. Never heard of Ebola
   e. Declined to respond

9. **Would you be willing to ride in an ambulance if you had a high fever today?***
   (single selection; prompted)
   a. Yes
   b. Unsure
   c. No
   d. Declined to respond

10. **Whom would you seek care from first if you had a high fever today?***
   (single selection; prompted)
   a. No one from outside the home / home-based care
   b. Traditional healer/spiritual healer
   c. Healthcare worker (e.g. doctor, nurse)
   d. Community health worker (e.g. Village Health Team; VHT)
   e. Pharmacist
   f. Other (specify):___________________________

g. Declined to respond

EBOLA AWARENESS

[I will ask you some questions about Ebola.]

11. **Have you heard of any place where people are getting infected with Ebola right now?***
(single selection; prompted)
    a. Yes → Go to Q12
    b. No → Go to Q13
    c. I don't know / not sure → Go to Q13
    d. I have never heard of Ebola before this interview → Go to Q13
    e. Declined to respond → Go to Q13

12. **From what you have heard, where are people getting <u>infected</u> with Ebola right now?***
(multiple selection; do not prompt)
    a. None
    b. Democratic Republic of Congo
    c. Uganda
    d. South Sudan
    e. Sierra Leone
    f. Liberia
    g. Guinea
    h. No response

13. **In which district in Uganda, if any, did some people get infected with Ebola in June 2019?***
(multiple selection; do not read choices)
    a. No district in Uganda had cases of Ebola
    b. Kisoro
    c. Kasese
    d. Arua
    e. Kampala
    f. Lamwo
    g. Busia
    h. I don't know / not sure
    i. Declined to respond

OVERALL EBOLA SENTIMENTS

<u>Interviewer to read to interviewee</u>: I will now ask you to tell me if you agree or disagree with a number of ideas, thoughts or beliefs about Ebola I will read to you. Please remember there is no right or wrong answer; we are only trying to find out what the community thinks and how people feel in order to keep improving Uganda's and Ugandans' performance in preventing and controlling this outbreak.

Supplement to: Musaazi J, Namageyo-Funa A, Carter VM, et al. Evaluation of community perceptions and prevention practices related to Ebola virus as part of outbreak preparedness in Uganda, 2020. *Glob Health Sci Pract*. 2022;10(3):e2100661. https://doi.org/10.9745/GHSP-D-21-00661

14. **Do you agree or disagree that Ebola is a man-made disease**?*
    (single selection; prompted)
    a. Agree
    b. Disagree
    c. Don't know / not sure
    d. Declined to respond

15. **Do you agree or disagree that if cases of Ebola start happening in Uganda, your community will fully cooperate with the authorities to stop Ebola from spreading.***
    (single selection; prompted)
    a. Agree
    b. Disagree
    c. Don't know / not sure
    d. Declined to respond

16. **Do you agree or disagree that if cases of Ebola start happening in Uganda, you and your family will fully cooperate with the authorities to stop Ebola from spreading.***
    (single selection; prompted)
    a. Agree
    b. Disagree
    c. Don't know / not sure
    d. Declined to respond

17. **Do you agree or disagree that if cases of Ebola start happening in Uganda, there is nothing someone can do to prevent Ebola from spreading?***
    (single selection; prompted)
    a. Agree
    b. Disagree
    c. Don't know / not sure
    d. Declined to respond

18. **Do you agree or disagree that if cases of Ebola start happening in Uganda, the bodies of people who may die from Ebola will be handled with respect by the authorities**?*
    (single selection; prompted)
    a. Agree
    b. Disagree
    c. Don't know / not sure
    d. Declined to respond

Supplement to: Musaazi J, Namageyo-Funa A, Carter VM, et al. Evaluation of community perceptions and prevention practices related to Ebola virus as part of outbreak preparedness in Uganda, 2020. *Glob Health Sci Pract*. 2022;10(3):e2100661. https://doi.org/10.9745/GHSP-D-21-00661

EBOLA RISK PERCEPTION

[Next I will ask you some questions about how you view your risk of getting Ebola]

19. What level of risk do you think you have in getting Ebola in the next 6 months?
(single selection; prompted)

a. No risk → Go to Q21
b. Small risk → Go to Q20
c. Moderate risk → Go to Q20
d. Great risk → Go to Q20
e. I don't know / not sure → Go to Q22
f. Declined to respond → Go to Q22

20. What is the <u>MAIN</u> reason for which you think you are at risk of getting Ebola?**
(single selection; do not read choices)

a. I have been experiencing signs and symptoms of Ebola
(if so: stop the interview, recommend that the person goes to the nearest health facility, provide the Ebola hotline phone number, record the address)
b. Someone in my family/household/dwelling may have Ebola
(if so: stop the interview, finding out if the person is still in the dwelling, recommend that the person goes to the nearest health facility, provide the Ebola hotline phone number, record the address)
c. I travelled to DRC recently
d. I am a health care professional
e. I live in the same household with a health care professional
f. I eat bush meat /hunt bush meat as my means of livelihood
g. Ebola is everywhere
h. I washed/touched the dead body of someone suspected/confirmed to have had Ebola
i. I have attended a burial/funeral ceremony of someone suspected/confirmed to have had Ebola
j. I may get Ebola from mosquito bites
k. I may get Ebola through the air
l. I have unprotected sex with someone who has survived Ebola
m. Others______________________________
n. I don't know / not sure
o. Declined to respond

Supplement to: Musaazi J, Namageyo-Funa A, Carter VM, et al. Evaluation of community perceptions and prevention practices related to Ebola virus as part of outbreak preparedness in Uganda, 2020. *Glob Health Sci Pract*. 2022;10(3):e2100661. https://doi.org/10.9745/GHSP-D-21-00661

21. What is the MAIN reason for which you do not think you are at risk of getting Ebola?**
(single selection; do not read choices)

a. Ebola is only a DRC problem
b. I do not eat or hunt bush meat or bats
c. I am not a health care or medical professional
d. I am a clean person / Ebola only affects unclean people
e. I don't live in an area where there is Ebola
f. I don't come in contact with someone with Ebola
g. God is protecting me
h. I have traditional powers that protect me from Ebola
i. I do not participate in burial ceremonies that involve the handling (touching/washing) of the dead body
j. I avoid all funerals and burials
k. I avoid unprotected contact with bodily fluids
l. I wash my hands with soap or other disinfectants
m. I do not have unprotected sex with someone who has survived Ebola
n. Others____
o. I don't know / not sure/
p. Declined to respond

EBOLA KNOWLEDGE

[Next I will ask you some questions about what causes Ebola, how people get it, ways to prevent it, and what to do if Ebola is suspected]

22. Please tell me what you think are all the possible causes of Ebola?**
(multiple selection; do not read choices)

a. Virus
b. Bats / Monkeys / Chimpanzees / Other wild animals
c. God or higher power
d. Witchcraft
e. Evildoing / Sin
f. Curse
g. Man-made
h. Others ____________________________________
i. I don't know/ not sure
j. Declined to respond

Supplement to: Musaazi J, Namageyo-Funa A, Carter VM, et al. Evaluation of community perceptions and prevention practices related to Ebola virus as part of outbreak preparedness in Uganda, 2020. *Glob Health Sci Pract*. 2022;10(3):e2100661. https://doi.org/10.9745/GHSP-D-21-00661

23. Please tell me what you think is the ways by which a person can get Ebola?**
(multiple selection; do not read choices)

a. By air
b. Mosquito bite
c. Bad odor or smell
d. Preparing bush meat as a meal (such as chimpanzees, monkeys, and other wild animals)
e. Eating bush meat
f. Eating fruits likely to have been bitten by bats
g. Saliva of an infected person
h. Blood of an infected person
i. Sweat of an infected person
j. Urine of an infected person
k. Feces of an infected person
l. Breast milk of an infected person
m. Sperm or vaginal fluid of an infected person
n. Shaking the hands of an infected person
o. Other physical contact with an infected person
p. God's will
q. Witchcraft
r. Others (specify): ________________________________
s. I don't know / not sure
t. Declined to respond

24. Can you please tell me what are the signs and symptoms of someone infected with Ebola?**
(multiple selection; do not read choices)

a. Any Fever
b. Sudden onset of high fever
c. Severe headache
d. Muscle pain
e. Weakness
f. Diarrhea (with or without blood)
g. Vomiting (with or without blood)
h. Abdominal (stomach) pain
i. Lack of appetite
j. Sore throat
k. Rash
l. Difficulty breathing
m. Bleeding (internal or external)
n. Others (specify):________________________________
o. I don't know / not sure
p. Declined to respond

25. **Do you believe that traditional healers can treat Ebola successfully?****
    (single selection; prompted)
    a. Yes
    b. No
    c. I don't know / not sure
    d. Declined to respond

26. **Do you believe that spiritual healers can treat Ebola successfully?****
    (single selection; prompted)
    a. Yes
    b. No
    c. I don't know / not sure
    d. Declined to respond

27. **Can someone prevent getting Ebola by avoiding mosquito bites?***
    (single selection; prompted)
    a. Yes
    b. No
    c. I don't know / not sure
    d. Declined to respond

28. **Can someone prevent getting Ebola by avoiding funeral or burial rituals that require handling the body of a person who has died from Ebola?****
    (single selection; prompted)
    a. Yes
    b. No
    c. I don't know / not sure
    d. Declined to respond

29. **If a person has Ebola, do you think he/she will have a greater chance of survival if he/she goes immediately to a health facility?****
    (single selection; prompted)
    a. Yes, she will have a greater chance of survival
    b. No, she will not have a greater chance of survival
    c. I don't know / not sure
    d. Declined to respond

30. **If a person with Ebola goes immediately to a health facility, do you think he/she will reduce the chance of spreading it to family/people living with them?****
    (single selection; prompted)
    a. Yes
    b. No

c. I don't know / not sure
d. Declined to respond

31. Do you think it is possible to survive and recover from Ebola?**
(single selection; prompted)
a. Yes
b. No
c. I don't know / not sure
d. Declined to respond

32. Once a man has survived Ebola, do you think he should use a condom during sex?*
(single selection; prompted)
a. Yes; **for how long? ____________________** **
b. No
c. It does not matter
d. I don't know / not sure
e. Declined to respond

33. Do you know of any number to call to report a suspected Ebola patient or death?
(single selection; prompted)
a. Yes, (33b) number:_______________
b. No→ Go to QX
c. I don't remember/not sure → Go to QX
d. Declined to respond → Go to QX

PRACTICES & BEHAVIORAL INTENTIONS

[Next, I will ask you some questions to better understand about health issues in your household, how people in the household get care for health problems, and other practices at the household or community level]

34. Has anyone in this household, including you, been sick <u>during the past month</u>?**
(single selection; prompted)
a. Yes → Go to Q35
b. No → Go to Q38
c. I don't know / not sure →Go to Q38
d. Declined to respond → Go to Q38

35. What were the signs and symptoms of the sick household member(s)?**
(multiple selection; do not read choices)
a. Fever
b. Headache
c. Muscle pain
d. Weakness

e. Diarrhea (with or without blood)
f. Vomiting (with or without blood)
g. Abdominal (stomach) pain
h. Lack of appetite
i. Sore throat
j. Rash
k. Difficulty breathing
l. Bleeding (internal or external)
m. Chest pain
n. Coughing
o. Others (specify)____
p. Don't remember
q. Declined to respond

36. Did the sick household member(s) go to a hospital or health facility?**
(single selection; prompted)
a. Yes → Go to Q38
b. No → Go to Q37
c. I don't know / not sure→ Go to Q38
d. Declined to respond → Go to Q38

37. What were the reasons for not going to a hospital or health facility?*
(multiple selection; not read choices)
a. Had no money / can't afford to pay
b. Believed the hospital/health facility could not help
c. Preferred to go to a nearby pharmacy instead
d. Preferred to go to a traditional or spiritual healer
e. Other_______________
f. Don't know / not sure
g. No response

38. In the past month, have <u>YOU</u> participated in a funeral/burial ceremony?
(single selection; prompted)
a. Yes → Go to Q39-42
b. No → Go to Q43
c. Declined to respond → Go to Q43

39. If yes, where did the funeral/burial ceremony take place?
(single selection; prompted)
a. In my current district in Uganda
b. Outside of my current district but within Uganda
c. Outside of Uganda (specify country:) ______________________
d. Declined to respond

40. If yes, where did people who attended the funeral/burial come from?*
(Multiple selection; prompted)
a. Everyone came from within my current district in Uganda
b. Some people came from other districts in Uganda
c. Some people came from DRC
d. Other (specify): ________________________________
e. I don't know / remember
f. Declined to answer

41. If yes, what happened at the funeral/burial?*
(multiple selection; do not read choices)
a. Religious leader prayed for the deceased
b. Family members observed burial from a distance
c. Traditional rituals involving physical contact with the corpse were performed
d. Attendees touched each other (hug, shake hands, etc.)
e. None of the above
f. Other (specify): ___________________________
g. I don't remember
h. Declined to respond

42. During the funeral / burial ceremony did YOU have any physical contact with the dead body?**
(single selection; prompted)
a. Yes
b. No
c. I don't remember
d. Declined to respond

43. Since you heard of Ebola, have you taken any action to avoid being infected?**
(single selection; prompted)
a. Yes → Go to Q44-45
b. No → Go to Q46
c. I don't know / can't remember → Go to Q46
d. Declined to respond → Go to Q46

44. In what ways have you changed your behavior or taken actions to avoid being infected? **
(multiple selection; do not read choices)
a. I wash my hands with soap and water more often
b. I wash my hands with just water more often
c. I clean my hands with other disinfectants more often
d. I try to avoid crowded places

e. I drink a lot of water / juice
f. I drink traditional herbs
g. I take antibiotics (e.g. penicillin, amoxilin)
h. I wear gloves (if so ask, how many times you change the gloves daily:_________)
i. I try to avoid physical contact with people I suspect may have Ebola
j. I avoid physical contact with everyone
k. I do not participate in burial ceremonies that involve the handling (touching/washing) of the dead body
l. I wash with salt and hot water
m. I use a condom when having sex with someone who has survived Ebola
n. I always use a condom when having sex
o. Others____
p. I don't know / not sure
q. Declined to respond

45. Of the actions you have taken to avoid being infected with Ebola, which one do you think is the most important?*

(multiple selection; do not read choices)

a. I wash my hands with soap and water more often
b. I wash my hands with just water more often
c. I clean my hands with other disinfectants more often
d. I try to avoid crowded places
e. I drink a lot of water / juice
f. I drink traditional herbs
g. I take antibiotics (e.g. penicillin, amoxilin)
h. I wear gloves (if so ask, how many times you change the gloves daily:_________)
i. I try to avoid physical contact with people I suspect may have Ebola
j. I avoid physical contact with everyone
k. I do not participate in burial ceremonies that involve the handling (touching/washing) of the dead body
l. I wash with salt and hot water
m. I use a condom when having sex with someone who has survived Ebola
n. I always use a condom when having sex
o. Others (specify):_____________________________________
p. I don't know / not sure
q. Declined to respond

46. What would you do if you suspect someone in your family has Ebola?**

(multiple selection; do not read choices)

a. Nothing
b. Report to district heath authorities
c. Help care for the person at home

d. Check the person's temperature by touching their body
e. Avoid all physical contact and bodily fluids of that person
f. Take the person to a health facility
g. Hide the person
h. Others (specify): ______________________________
i. I don't know / not sure
j. Declined to respond

47. What do you think health workers would do if someone suspected of having Ebola goes to the hospital / health facility?**
(multiple selection; do not read choices)
a. They won't be able to do anything for him/her and may die there
b. They will take care of him/her
c. They will definitely cure the person from Ebola
d. They will find a way to kill the patient
e. They turn the patient away
f. Others (specify): ______________________________
g. I don't know / not sure
h. Declined to respond

48. How important is it for family members to be able to visit and see a relative who is admitted to a health facility due to Ebola?*
(single selection; prompted)
a. Important
b. Somewhat important
c. Not important
d. Declined to respond

49. If a family member died, would you accept alternatives to traditional funeral/burial that would NOT involve the touching or washing of the dead body?**
(single selection; prompted)
a. Yes
b. No
c. I don't know / not sure
d. Declined to respond

EBOLA VACCINATION

[Next I will questions about Ebola vaccines.]

50. Have you heard about an Ebola vaccine <u>before this interview</u>?*
(single selection; prompted)
a. Yes → Go to Q51-57
b. No → Go to Q58
c. I don't know/remember → Go to Q58
d. Declined to respond → Go to Q58

51. Please say if you agree, somewhat agree, disagree, or have no opinion about the following statement: "If Uganda started having cases of Ebola, an Ebola vaccine is needed to help prevent the spread of the disease in the country"*
(single selection; prompted)
a. Agree

b. Somewhat agree
c. Disagree
d. I don't know / I'm not sure
e. Declined to respond

52. **If there is an Ebola outbreak in your district, who do you think should be the first to get an Ebola vaccine?****
(single selection, do not read choices)
a. Me/my family
b. Healthcare workers
c. Burial teams
d. Political leaders
e. Pregnant women
f. Children
g. Team that is offering the Ebola vaccine
h. People who live in worst affected areas
i. Other: ______________________________
j. No one should get the vaccine
k. I don't know /no opinion
l. Declined to answer

53. **If there is an Ebola outbreak in your district, how many people in your community do you think would agree to take an Ebola vaccine if they were offered it?***
(single selection; prompted)
a. No one
b. Some people
c. Most people
d. Everyone
e. I don't know / no opinion
f. Declined to respond

54. **If there is an Ebola outbreak in your district, how likely would you be to take an Ebola vaccine for yourself if you were offered it?***
(single selection; prompted)
a. Very likely to take it
b. Somewhat likely to take it
c. Not very likely to take it
d. Not at all likely to take it
e. I don't know / I'm not sure
f. Declined to answer

55. **What concerns do you have about taking an Ebola vaccine?***
(multiple selection; do not read choices)
a. I have no concerns about the Ebola vaccine

b. It may cause Ebola
c. It may cause death
d. It may cause side effects like muscle aches and body pain in the short term
e. It may cause infertility or impotence/sexual weakness
f. It cannot prevent Ebola
g. Lack of trust in vaccine manufacturer
h. Lack of trust in the process used to make the vaccine
i. Lack of trust in the team offering the vaccine
j. Lack of trust in the health system
k. Other:________________________
l. I don't know / I'm not sure
m. Declined to respond

56. Have you ever been offered an Ebola vaccine?*
(single selection; prompted)
a. Yes → Go to Q57
b. No → Go to Q58
c. Declined to respond → Go to Q58

57. Did you take the vaccine when it was offered to you?*
(single selection; prompted)
a. Yes
b. No
c. Declined to respond

STIGMA & DISCRIMINATION

58. Would you buy fresh vegetables from a shopkeeper who survived Ebola and has a certificate from a Government Health Facility stating he/she is now Ebola-free?*
(single selection; prompted)
a. Yes
b. No
c. I don't know / not sure
d. Declined to respond

59. Do you think that a school pupil who has survived Ebola and has a certificate from a Government Health Facility stating he/she is Ebola-free puts other pupils in their class at risk of infection?*
(single selection; prompted)
a. Yes
b. No
c. I don't know / not sure

d. Declined to respond

60. Would you welcome someone back into your community/neighborhood after he/she has recovered from Ebola?*
(single selection; prompted)
a. Yes
b. No
c. I don't know / not sure
d. Declined to respond

INFORMATION SOURCES & EXPOSURE

61. Which Ebola prevention message(s) have you received in the past 6 months?*
(multiple selection; do not read choices)
a. I haven't received any Ebola messages → Go to Q63
b. Avoid participating in funeral practices and traditional burials that involve contact with the corpse
c. Avoid contact with sick people who have Ebola-like symptoms
d. Report deaths that resemble Ebola to health authorities
e. Report sick people to health authorities
f. Wash your hands
g. Do not eat bush meat
h. Other (specify): ______________________________
i. Declined to respond

62. How did you receive the Ebola message?*
(multiple selection; do not read choices)
a. Radio
b. Television
c. Megaphone public announcements
d. Household visit by a health worker or other Ebola response worker
e. Church / Mosque / other religious venues
f. Other community meetings
g. Film vans
h. Newspaper /Newsletter/ Other print materials
i. Posters / flyers
j. Internet / Blog / Website / Social Media / Facebook
k. Mobile phone / text messages / WhatsApp
l. Traditional leaders (chief, village headman, etc.)
m. Ministry of Health and Sanitation
n. Informational visits to the school I work at or attend
o. Other (specify): ______________________________
p. I don't know / not sure
q. Declined to respond

Supplement to: Musaazi J, Namageyo-Funa A, Carter VM, et al. Evaluation of community perceptions and prevention practices related to Ebola virus as part of outbreak preparedness in Uganda, 2020. *Glob Health Sci Pract*. 2022;10(3):e2100661. https://doi.org/10.9745/GHSP-D-21-00661

63. What topic related to Ebola would you like more information on?
(multiple selection; do not read choices)
a. I don't want any more information on Ebola
b. Ebola outbreak in DRC
c. Sign sand symptoms of Ebola
d. How Ebola is spread
e. How Ebola can be prevented
f. Where people can go to get treatment for Ebola
g. How people who get Ebola can be cared for
h. How people who get Ebola can be safely buried
i. Monitoring of people who come in contact with an Ebola patient
j. Ebola vaccine safety
k. Ebola vaccine duration of protection
l. Ebola vaccine eligibility
m. Ebola survivors transmission of Ebola through casual contact
n. Ebola survivors transmission of Ebola through sexual contact
o. Other (specify): ____
p. Declined to respond

TRAVEL & VISITATIONS

[We are now almost at the end of the interview. I'd like to ask you about places you have travelled to or have people visit you from.]

64. In the past 6 months, did you travel to a place outside your district?**
(single selection; prompted)
a. Yes → Go to Q65
b. No → Go to Q66
c. Declined to respond → Go to Q66

65. Where did you travel to?**
(multiple selection; do not read choices)
a. Within Uganda (specify district):__________*(use dropdown list in tablet)*
b. Democratic Republic of Congo (DRC)
c. South Sudan (specify):__________________ *(use dropdown list in tablet)*
d. Kenya (specify):_______________________ *(use dropdown list in tablet)*
e. Rwanda (specify):______________________ *(use dropdown list in tablet)*
f. Kenya (specify):_______________________ *(use dropdown list in tablet)*
g. Other (specify): _______________________
h. Declined to respond

Supplement to: Musaazi J, Namageyo-Funa A, Carter VM, et al. Evaluation of community perceptions and prevention practices related to Ebola virus as part of outbreak preparedness in Uganda, 2020. *Glob Health Sci Pract*. 2022;10(3):e2100661. https://doi.org/10.9745/GHSP-D-21-00661

66. **In the past 6 months, did someone from a place outside your district traveled to visit your or another household member****
    (single selection; prompted)
    a. Yes → Go to Q67
    b. No → Go to CLOSING script
    c. I don't know / not sure → Go to Q68
    d. Declined to respond → Go to Q68

67. **Where did the person(s) traveled from?****
    (multiple selection; do not read choices)
    a. Within Uganda (specify district):__________ *(use dropdown list in tablet)*
    b. Democratic Republic of Congo (DRC)
    c. South Sudan (specify):____________________ *(use dropdown list in tablet)*
    d. Kenya (specify):_________________________ *(use dropdown list in tablet)*
    e. Rwanda (specify):________________________ *(use dropdown list in tablet)*
    f. Kenya (specify):_________________________ *(use dropdown list in tablet)*
    g. Other (specify): _________________________*(use dropdown list in tablet)*
    h. Declined to respond

INVESTIGATION THERAPEUTICS

68. ***Imagine your relative had Ebola, and there was a medical treatment that has shown to be safe when used in humans but it is unknown if the treatment actually works.***
    Would you be willing to accept this treatment for your relative?*
    (single selection; prompted)
    a. Yes
    b. No
    c. I don't know / not sure
    d. Declined to respond

Supplement to: Musaazi J, Namageyo-Funa A, Carter VM, et al. Evaluation of community perceptions and prevention practices related to Ebola virus as part of outbreak preparedness in Uganda, 2020. *Glob Health Sci Pract*. 2022;10(3):e2100661. https://doi.org/10.9745/GHSP-D-21-00661

CLOSING SCRIPT

- *Thank you for taking the time to discuss these important issues with me.*
- *Again, please rest assured that your responses will be kept confidential.*
- *Your name or any other contact of your family or household will not be included in the report.*
- *The responses you have provided will help in improving risk communication activities and other efforts aimed at protecting the people of Uganda against Ebola.*
- *Once again, thank you very much.*

DO YOU HAVE ANY QUESTIONS FOR ME?

Instructions to Interview: Answer questions you are have been trained to answer, then inform participant that he/she can call the supervisor (information below) if they have additional questions about participating or they should consult the number on the Ebola flyer left behind if they have questions about Ebola.

Supervisor Name:____

Phone number: ____
