# Supplementary material for: Evaluation of Community Perceptions and Prevention Practices Related to Ebola Virus as Part of Outbreak Preparedness in Uganda, 2020
Source: Glob Health Sci Pract. 2022 Jun 29;10(3):e2100661. doi: 10.9745/GHSP-D-21-00661 (PMC9242603; doi:10.9745/GHSP-D-21-00661)
Supplement: GHSP-D-21-00661-supplement-1.pdf [file GHSP-D-21-00661-supplement-1.pdf]

Supplement to: Musaazi J, Namageyo-Funa A, Carter VM, et al. Evaluation of community perceptions and prevention practices related to Ebola virus as part of outbreak preparedness in Uganda, 2020. *Glob Health Sci Pract*. 2022;10(3):e2100661.
https://doi.org/10.9745/GHSP-D-21-00661

Protocol

Assessment of Public Knowledge, Attitudes, and Practices Related to Ebola Virus Disease Prevention and Treatment in Uganda

September 2019

Submitted to:

Science and Ethics Committee

Uganda Virus Research Institute

Submitted by:

Infectious Diseases Institute

College of Health Sciences, Makerere University, Uganda

Supplement to: Musaazi J, Namageyo-Funa A, Carter VM, et al. Evaluation of community perceptions and prevention practices related to Ebola virus as part of outbreak preparedness in Uganda, 2020. *Glob Health Sci Pract*. 2022;10(3):e2100661.
https://doi.org/10.9745/GHSP-D-21-00661

Supplement to: Musaazi J, Namageyo-Funa A, Carter VM, et al. Evaluation of community perceptions and prevention practices related to Ebola virus as part of outbreak preparedness in Uganda, 2020. *Glob Health Sci Pract*. 2022;10(3):e2100661.
https://doi.org/10.9745/GHSP-D-21-00661

Table of Contents

Background 4

Objectives 6

Methodology 6

Design 6

Sampling 6

Sample size calculations 7

Selection of clusters 7

Selection of households 8

Selection of interviewees 8

Implications of an EVD outbreak before or during data collection 10

Training of data collectors 10

Data collection 11

Use of digital technology in data collection 11

Data cleaning 12

Data analysis 13

Reporting of findings 13

Risks and benefits 13

Limitations 17

Annex 1: Estimated timeline of key activities 17

Annex 2: Investigators 19

Annex 3: Interview Consent Script 30

Annex 4: Participant Consent Form 31

Annex 5: Parental Consent Form 33

Annex 6: Questionnaire 35

References 59

Supplement to: Musaazi J, Namageyo-Funa A, Carter VM, et al. Evaluation of community perceptions and prevention practices related to Ebola virus as part of outbreak preparedness in Uganda, 2020. *Glob Health Sci Pract*. 2022;10(3):e2100661. https://doi.org/10.9745/GHSP-D-21-00661

Background

An outbreak of Ebola Virus Disease (EVD)[1,2] was detected by the Uganda Ministry of Health on 11th June 2019 in Kasese district in South Western Uganda[3]. The Kasese outbreak marked the sixth EVD outbreak in Uganda. The index case in Kasese was a young child that had recently traveled to the Democratic Republic of Congo (DRC) along with family members. After testing positive for the Zaire strain of Ebolavirus, the child subsequently died on 11th July 2019. Shortly thereafter the child's grandmother and younger brother also tested positive for EVD and died.[3]

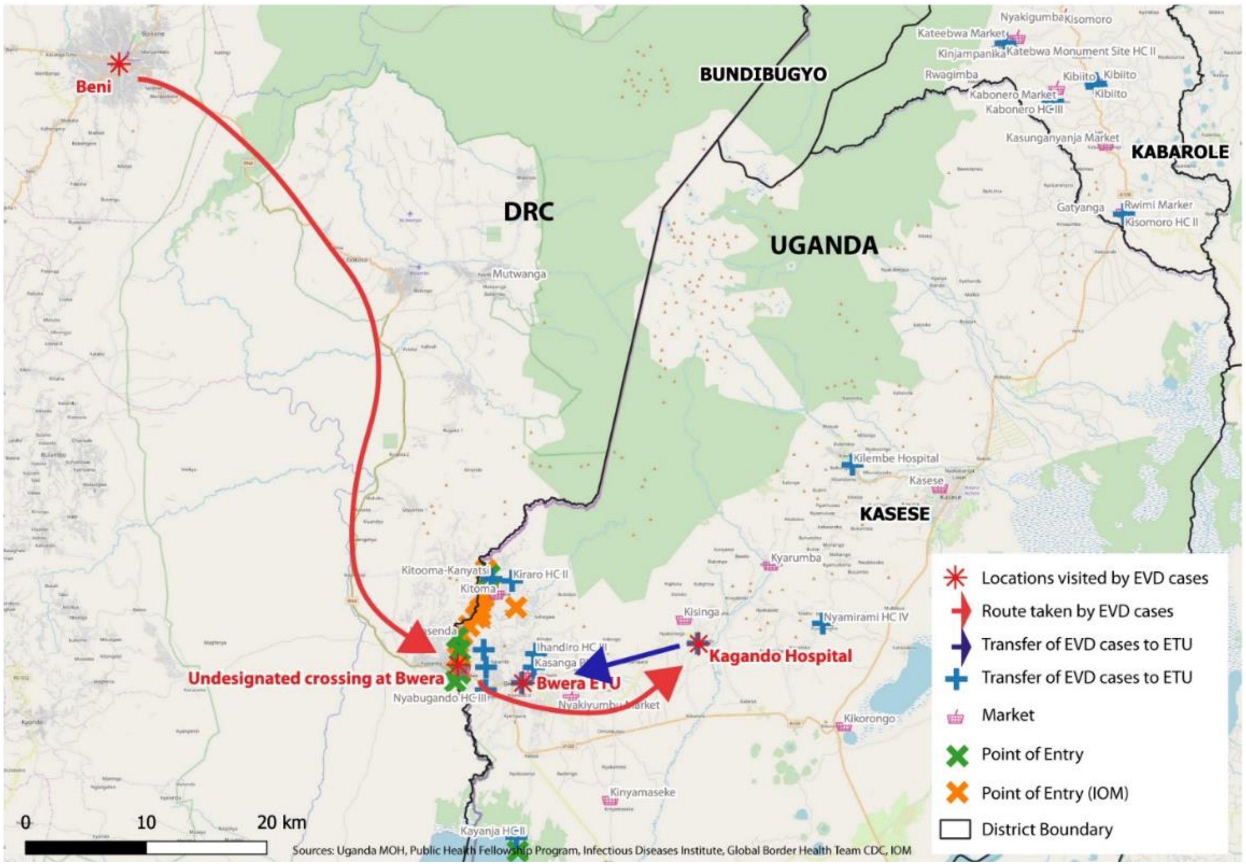

Figure 1. Movement of the three EVD cases from DRC into Uganda; data source: World Health Organization

The EVD outbreak in Kasese was promptly contained by the Uganda Ministry of Health and response partners constituting the National Task Force for Public Health Emergencies (NTF). Outbreak response strategies included identification of contacts of the EVD cases,

monitoring of contacts identified, and vaccinating of contacts. As of the last WHO SitRep available on July 15th 114 contacts were identified and 1507 cumulative individuals were vaccinated (78 contacts, 747 contact of contacts, and 682 frontline health workers)[3]

Despite the success of the Kasese response in the containing the outbreak, the importation of cases into Uganda is a stark reminder of the high risk posed by the second largest outbreak of EVD in DRC to Uganda's border districts with DRC. As part of preparedness efforts, understanding gaps in the public's knowledge, attitudes, and practices (KAP) related to EVD will help guide the development of evidence-based risk communication and social mobilization strategies and other interventions to help prevent the spread of the virus should it enter Uganda.

Changes to care seeking behaviors and burial rituals involving contact with corpses were critical to containing the 2014-2015 Ebola outbreak in West Africa.[4-12] Various KAP surveys were conducted during the West Africa outbreak[13-16] and current outbreak in DRC[17,18] that informed outbreak response and preparedness activities including implementation of risk communication and social mobilization.[19,20] Uganda will likewise benefit from having district-specific robust and reliable baseline estimates of important measures of EVD-related knowledge and misconceptions, social perceptions of survivors, EVD vaccine attitudes, intentions to seek EVD medical care, acceptance of safe burial measures, EVD sexual transmission risks, trust in health care delivery systems, and self-reported protective behaviors.

The United States Centers for Disease Control and Prevention (CDC) has been supporting the Government of Uganda to build capacity and strengthen health systems to prevent, detect, and respond to global health security threats.[21] In the context of the current epidemic in DRC and as part of EVD preparedness in Uganda, CDC is supporting the governments of DRC and Uganda to coordinate activities and provide technical assistance in numerous areas including laboratory testing, contact tracing, infection prevention control, border health screening, data management, risk communication, and vaccination. It is against this background that CDC funded the Infectious Diseases Institute (IDI) at Makerere University in Uganda to undertake an EVD geographic risk-focused KAP survey to inform evidence-based, data-driven strategies for

risk communication and social mobilization and other EVD interventions for use in both the current preparedness environment and possible future outbreak responses.

Objectives

1. Measure district-level estimates in six districts in Uganda of the public's knowledge, beliefs, attitudes, and practices related to Ebola Virus Disease (EVD) risks due to the ongoing and widespread EVD outbreak in DRC; and
2. Identify socio-behavioral and health system barriers that may hinder the containment of an EVD outbreak in Uganda given the complex socio-political and security context of the protracted outbreak in neighboring DRC.

Methodology

Design

The assessment will employ a cross-sectional household survey targeting 3,840 randomly selected respondents in order to better understand the public's knowledge, attitudes, and practices related to EVD in Uganda settings within the context of the current DRC outbreak. Six districts have been purposively identified for inclusion in the assessment based on their EVD risk profiles. Four high risk districts bordering DRC (Kasese, Kisoro, Arua, and Kampala) along with two low risk profile districts sharing borders with Kenya and South Sudan (Busia and Lamwo , respectively). The sample for this EVD KAP survey is powered to provide district-specific estimates of key indicators but not to produce national, regional, or rural-urban estimates of EVD-related KAP. Such designs would be costly and outside the scope of the objectives of this KAP survey.

Sampling

In each of the selected districts, a stage sampling approach will be used to select clusters, households, and individuals. At the first stage, primary sampling units (clusters) will be

randomly selected from a sampling frame. In the second stage, households will be selected using systematic random sampling. In the third and final stage, two eligible individuals will be selected from each household using quota sampling (head of household plus an additional household member).

Sample size calculations

For each district, minimum sample sizes have been calculated independently on the assumption that the prevalence of knowing at least one means of Ebola prevention is conservatively estimated at 50%, with 10% confidence interval width (precision), alpha set at 0.05 (95% confidence coefficient), design effect estimated to be ~4 (based on intra-class correlation coefficient of 0.10 and an average of 30 participants selected per cluster). This results in a minimum required sample of approximately 480 participants and 16 clusters per district in order to attain district-level estimates of KAP outcomes within the parameters outlined above. However, assuming 75% response rate on average, 640 eligible individuals from 20 clusters would therefore have to be approached for consent in each district (applying an inflation factor of 1.33 [1/0.75] to the required sample) (Table 1).

Analysis of the KAP data (including for district-level estimates), will require appropriate weighting of the data to account for the multi-stage cluster design. Inverse probability weights (the inverse probability of selection at each of the three levels), would be calculated and used for weighting the results. Because sample size calculations were estimated for each district separately to allow for district-level estimates, use of post-stratification weights will be necessary when pooling data from the six districts combined to calculate the pooled estimates of EVD-related knowledge, attitudes, and practices.

Selection of clusters

The Uganda National Population and Housing Census (UNPHC) conducted in 2014 will provide a sampling frame of enumeration areas (EAs) for each district that will be obtained from the Uganda Bureau of Statistics. Based on the most recent UNPHC listing of EAs, 20 EAs (clusters) will be selected from each district using probability proportional to size. This means that clusters with larger population size will have greater probability of selection compared to

smaller clusters. According to the UNPHC, an EA in Uganda is a geographic area that has about 130 households on average.

Selection of households

From the total number of households in the cluster, 16 households will be selected within each cluster using systematic random sampling whereby the households are selected as follows:

- Enumerating every household in the cluster to get a denominator of households;
- Calculating a sampling interval for the cluster (k);
    - k = total number of households in the cluster divided by 16
- Randomly selecting a household to approach for the first interview; and
- Subsequent selecting households to approach based on sampling interval until 16 households have been approached for their consent and potential participation.

In each selected cluster, the district coordinator and team supervisors will first engage the district health teams and local community leaders ahead of data collection to make them aware of the activity and reduce the risk of community suspicions and/or misinformation regarding the purpose of the activity. Each household will be visited up to three times (on separate days) in the same week as part of recruitment efforts in that cluster. However, non-responsive households or households that decline to participate will not be replaced to the extent that the sample has been adjusted to accommodate up to 25% non-responsiveness across clusters.

Selection of interviewees

Interviews will be conducted with two individuals from each randomly selected household. The household head will always be selected and approached for consent and participation given his/her influential role on the decisions and practices within the household. It is estimated that about one-third of households are headed by a female in Uganda.[1] Therefore, anticipating that a substantial proportion of household heads would be older men, a second randomly selected household member will be approached for consent and potential participation. To be eligible, the second member will be either a woman age 25 years and above or any other household

[1] World Bank. https://data.worldbank.org/indicator/SP.HOU.FEMA.ZS

member between the ages of 15 and 24 years. This approach will allow for diversity in the sample to sufficiently include women and adolescents/youths. Otherwise, the sample would risk being biased towards household heads.

All participants approached for the survey (regardless of their age) will need to provide individual written consent in order for the interview to be initiated. Respondents between 15-17 years are important to include in the survey because they make up a substantial proportion of the Uganda population, and carry a potential EVD risk if there is an outbreak. In the DRC outbreak for example, about a quarter of the confirmed cases have been young people less than 18 years old. Therefore, it is important to understand this group's EVD-related knowledge, attitudes, and practices related in order to inform interventions to help reduce their EVD risk. For these group of target participants ages 15-17 years, consent shall be sought from one of the parents. If none of the parents is available, then consent from the child's guardian or household head will be sought. In addition, assent will be sought from the child directly. It should be noted that a child's dissent will take precedence over consent from his/her parent, guardian, or head of household.

If a participant is not able to read and write, eligible participants will be asked to sign the consent form with their thumb-print. No interview will be conducted without the written or thumbprint signed consent of the prospective participant. The consent form that will be used in recruiting participants is provided in Annex 3. Two copies of the consent form must be signed by each participant – one will be left with the participant and the second will be kept by the survey team. All consent forms will be in paper format. Signed consent forms will be submitted to team supervisors at the end of each day of data collection. District coordinators will collect signed consent forms from team supervisors at least twice a week. District supervisors will store the signed consent forms in a secured box with lock and key, for final submission to the IDI project manager at the end of data collection. The project manager will ensure secured storage of the signed consent forms in an access-controlled room in the IDI office, separately from where respondent's questionnaire responses are stored, managed, processed, or analyzed. Financial incentives will not be provided to participants as part of the recruitment process. Instead, a bar

of soap and an EVD flyer with key prevention and safety messages will be provided to the household at the end of the interview in recognition of the time spent by participants and to help reinforce protective behavior. If the household does not have any literate member, data collectors will verbally explain the information on the flyer.

Implications of an EVD outbreak before or during data collection

In the event an Ebola outbreak is declared in Uganda before or during data collection, the principal investigator will contact the ethics committee. If the outbreak occurs in one or more of the existing districts where data collection is happening for the KAP assessment, data collection teams in said district(s) will receive a refresher training on safety and security procedures as outlined in Table 2. In the event that an outbreak were to occur in a district outside where data collection is not happening for the KAP assessment, the PI and co-investigators will consult with the Uganda MoH to decide on adding said district(s) into the sampling frame. In such scenario, it would be an ethical public health priority to include outbreak areas in the KAP assessment to help inform the response activities for risk communication and social mobilization.

Training of data collectors

Based on the current plan, IDI will recruit and train 36 data collectors in total (6 per district) and 12 team supervisors (2 per district) on the survey protocol instrument during a five-day workshop. Data collection teams will have an opportunity to pilot the questionnaire in a conveniently selected community in the district where they will be assigned. These pilot communities will be selected from the list of clusters that are not included in survey. Feedback from the pilot will be used to refine items on the questionnaire.

The training will focus on the following six core areas:

- Overall assessment protocol
- Informed consent
- Safety and security precautions
- Cultural context of selected districts

- Administration of questionnaire
- Quality control and assurance

Data collectors will be recruited partly based on their ability to speak the predominant local language(s) of their assigned districts in addition to basic secondary school ordinary level certificate completion or equivalent. Supervisors will be required to have at least a bachelor-level degree in a relevant health or social science discipline. The trained data collectors and supervisors will be subsequently divided into respective teams. Each team comprises 3 data collectors and 1 supervisor. Each team will then be assigned to specified geographic clusters within their assigned districts. Data collection for each KAP survey is expected to last for a total of approximately 15-20 days in each district. Data collection will be done concurrently across districts. It is anticipated that each interview will take about 50-60 minutes to fully administer.

Data collection

Trained data collectors will be responsible for administering the questionnaire to eligible and consented participants. The team supervisors will oversee the day-to-day activities of data collectors, and provide on-the-ground support supervision to ensure maximal quality control and quality assurance as per protocol. CDC staff will provide technical support and serve in an advisory role but will not be involved in conducting interviews.

Use of digital technology in data collection

Digital data collection will be done using computer tablets programmed with the questionnaire items to enhance overall data quality, ensure rapid collection of the data, and reduce the need for double-entry verifications when done using paper-based questionnaires. A mirror of the questionnaire will be programmed using Open Data Kit (ODK) and subsequently installed and configured on the mobile devices prior to the training of data collectors. The ODK questionnaire will be updated to reflect feedback from the pilot during the training of data collectors. A finalized and tested version of the ODK questionnaire will be deployed prior to data collection.

Data submitted from the tablets through ODK will be securely stored locally on the tablet and submitted to a secured web-based hosting server. Team supervisors will be responsible for synching the devices with the hosting server at the end of each day.

Global Positioning System (GPS) coordinates of households visited will be captured as part of the ODK questionnaire for quality assurance purposes. Only the project manager and data managers from IDI will be able to access the secured password protected hosting server where the GPS data and submitted interview data are stored. A real-time map of the household GPS data points collected will be automatically generated by the web-based hosting platform to enable data managers on the project to review if data collectors are capturing households with a plausible geographic spread that indicate random selection as per protocol. For instance, very close clustering of households on the map would be an indication that the random selection process may have not been properly implemented as per protocol. In such scenario, the project manager will communicate those observations with district coordinators and team supervisors for corrective action by the associated team to ensure appropriate random selection of households as per the established steps in this protocol.

The computers used to access any of the digitally stored data will be password protected, and will only be accessed by the project manager and designated data manager supporting the project. Files downloaded from the hosting server will first be stored onto a designated encrypted password-protected computer physically located in a secured location in the IDI office. Only the project manager and designated data managers will have access to the computer storing the downloaded dataset. Once data collection has been completed, variables with geo-location data will be removed from the dataset before sharing the dataset with any data analysts.

Data cleaning

A designated data manager will perform quality assurance checks during the data collection period to identify any potential data quality issues and come up with corrective actions. After data collection, any necessary data cleaning will be performed by the data manager with support from other designated data analysts from IDI and/or CDC.

Data analysis

The data collected through ODK will be exported into a CSV or XLS file, and then further imported into a statistical analysis software such as Stata, SAS, or SPSS for processing, management, and analysis. The first phase of data analysis will mainly comprise descriptive analysis of key variables of interest measuring core KAP indicators in order to fulfill objectives 1 and 2 of the assessment. Bivariate analyses will be undertaken to compare differences in measures of Ebola KAP between and across districts. In addition, differences in KAP outcomes will be assessed based on the EVD risk profiles of the districts (low, medium, high). In the second phase of the analysis, multivariable analysis will be undertaken to identify determinants of EVD knowledge, attitudes, and practices in the context of outbreak preparedness. Additional qualitative analysis may be conducted on narrative responses that help interpret quantitative outcomes, responses patterns and open-ended responses.

Reporting of findings

Findings from the first phase of the analysis will feed into the development of a preliminary report that will be shared with the Risk Communication and Social Mobilization Committee and other EVD committees of the NTF to inform preparedness efforts. A special presentation will be made to the EVD National Taskforce for further review and approval. IDI and CDC will develop a final report based on the various analyses and with input from relevant partners, including MoH, UNICEF, and WHO.

Risks and benefits

The overall risk posed to participants in the assessment is minimal. Potential risks to participants or data collectors that may arise during or after the assessment are described in Table 2. For each risk identified, mitigation steps to minimize the risk and its impact are described. While participants will not directly benefit from the assessment, the collective insights gleaned from their responses will inform data-driven interventions that can be targeted for EVD preparedness and potential outbreak response in their districts and Uganda as a whole – especially for risk

communication and behavior change efforts. Health system barriers will also be identified for improvements to foster greater community cooperation.

Table 2. Risk assessments and mitigation measures

**Risk**	**Likelihood**	**Impact**	**Mitigating Strategy**
If an outbreak is declared: Exposing data collection teams to households with possible Ebola cases	Low	High	Adequately train data collection teams on established safety and security when interacting with household members; provide on-the-ground supervision at district level; immediately take corrective measures to address breach in protocol; monitor data collectors in high EVD risk areas for signs and symptoms of EVD.<br><br>Note: Data collection teams will be trained to avoid physical contact with participants and objects within and around the households. Interviews will be conducted outside of the dwelling (front entrance or veranda for example) with appropriate social distancing according to WHO recommendations. No interviews will be conducted inside the dwelling/house.
If an outbreak is declared: Exposing respondents to Ebola through physical contact with data collection teams	Low	High	
Revealing the identity of participants	Low	Medium	Adequately train data collection teams on informed consent procedures; ensure that informed consent forms are signed or thumb-printed for each interview; supervise and monitor data collection

			teams to ensure they are adhering to the informed consent procedures as per protocol. Names of respondents will never be collected in the questionnaire. Consent forms will remain in paper-based format and stored separately from the completed digital questionnaires. Any personal identifying information collected on signed consent form will always be stored separately and not linked to the questionnaire of that particular participant.
Communities reject data collectors and become uncooperative	Low	Low	In each enumeration location, data collection teams will seek the approval of the community leaders (such as village chiefs) before undertaking any project activity. The local councilors and district health teams will also be advised of the data collection efforts in their respective localities. If the safety and wellbeing of any of the data collection teams is threatened, then IDI will randomly select a substitute cluster within the district using the sampling procedures established in the protocol.
Violence or threats of violence towards	Low	High	Threats of violence by communities against health workers and other mobilisers have been documented in the

data collection teams			current DRC outbreak and the 2014-2015 EVD outbreak in West Africa. All data collection teams will be subjected to thorough safety and security rules, including training on how to gain entry into communities coupled with the provision of free communication and emergency transportation means. Local district health teams will be informed of the activity to keep them appraised, and will be contacted if there are any issues of community resistance or violence toward data collection teams.<br><br>Where large scale civil unrest breaks out or pockets of violence occur for an extended period of time, IDI will terminate all data collection efforts in the said locality and find a suitable substitute either through random or purposive selection.
Selecting a household that lost a relative due to EVD	Low	Medium	Because of the low likelihood of occurrence, it is not deemed necessary to screen participants to determine if they have lost a family member or relative due to EVD. However, if it occurs and becomes known to the data collector at any given point before or during the interview, extra caution will be taken to

			ensure that the selected individual is comfortable to proceed with the interview. Specifically, the individual will be given another opportunity to opt out of the interview.

Limitations

The overall sample will contain proportionally more enumeration from districts classified as having high EVD risk due to the ongoing outbreak in neighboring DRC. These areas may have higher level of knowledge and better prevention practices relating to EVD as a result of their potential increased exposure to ongoing social mobilization and risk communication interventions as part of ongoing EVD outbreak preparedness. However, powering the survey to produce reliable estimates of the outcomes at district level helps to mitigate this possible limitation in how the results will the interpreted. Another key limitation is that self-reported practices may not always be aligned with individual's actual behaviors. It is therefore possible that respondents may provide socially desirable responses; especially due to their possible awareness of messages during the outbreak in Kasese and/or from the DRC outbreak. Finally, this KAP survey is not powered nor intends to compare results across sub-groups (e.g. by gender or age). Also, the results may not always be directly applicable to other health threats or potential outbreaks given the EVD focus of this survey.

Annex 1: Estimated timeline of key activities

**Activities**	**Timeline (2019)**
• Engagement of EVD response partners in protocol development<br>• Preparation and submission of IRB application	August
• Recruitment of project personnel<br>• Development of training materials	September

• Procurement of project materials, equipment, and services	
• Training of data collection teams and supervisors<br>• Pre-test data collection instruments and refine accordingly<br>• Data collection (tentative; dependent on ethical approval)<br>• Finalize data analysis plan	October
• Data cleaning, tabulation, and preliminary analysis<br>• Development and sharing of preliminary findings	November
• Additional analysis<br>• Finalizing and disseminating of report	December

Supplement to: Musaazi J, Namageyo-Funa A, Carter VM, et al. Evaluation of community perceptions and prevention practices related to Ebola virus as part of outbreak preparedness in Uganda, 2020. *Glob Health Sci Pract*. 2022;10(3):e2100661.
https://doi.org/10.9745/GHSP-D-21-00661

Annex 2: Investigators

Principal Investigator<br>Mohammed Lamorde, PhD, FRCP<br>Infectious Diseases Institute, Makerere University	
**IDI co-investigators**	**CDC co-investigators**
Joanita Kigozi, MRCP<br>Richard Walwema, BMLT, MBA<br>Apollo Olowo, MPH	Mohamed F. Jalloh, MPH<br>Vance Brown, MA<br>Victoria Carter, MPH, PhD<br>Rosalind Carter, PhD<br>Rose Apondi, MPH

Principal Investigator: Mohammed Lamorde, PhD, FRCP

Mohammed Lamorde PhD, FRCP is responsible for strategic development, staff development and training, of the Global Health Security Program at the IDI. Dr Lamorde has worked at the IDI since 2006 with experience spanning from clinical care, research and health systems strengthening. He is the Project Director of the Global Health Security Partner Engagement Project (Uganda) funded by the United States Centers for Disease Control and Prevention (1U2GGH001744-01) responsible for overall leadership and implementation of the project in over 20 districts of Uganda. Through this project, he works closely with the Ministry of Health in Uganda and other national stakeholders to support Uganda's effort to meet its International Health Regulations (2005) obligations in the following technical areas: biosafety and biosecurity, prevention of the emergence and spread of antimicrobial resistance; disease surveillance; and national laboratory system and emergency preparedness for viral hemorrhagic fevers. The EVD KAP Survey is funded through this grant mechanism.

Dr Lamorde serves as Project Director a project funded by Resolve to Save Lives aimed at accelerating progress on the implementation of the National Action Plan for Health Security. He is a co-investigator on the Joint Mobile Emerging Diseases Intervention Clinical Capability project, an international clinical research consortium funded by the United States Department of Defence to advance drug development (Phase II/III studies) for investigational therapeutics against filoviruses. He is Principal Investigator on the CAPA-CT and the CAPA-CT II projects that support drug development for therapeutics for filovirus infections and surveillance research for zoonotic infections.

Co-investigator: Joanita Kigozi MSc, MRCP

Dr Joanita Kigozi holds a Bachelor's Degree in Medicine and Surgery (MBChB) from Makerere University and is a member of the Royal College of Physicians (MRCP-UK). She also holds a Master's Degree in Infectious Diseases from the London School of Hygiene and Tropical Medicine as well as the Arthur Ashe Fellowship for International Health Care Workers (Cornell University, New York, USA).

Dr Kigozi Joined the IDI Outreach Department in 2010 as a Physician/HIV specialist and is currently the Deputy Head of the Outreach Department. She is also the project manager for the Infectious Diseases Institute- Kampala Region HIV capacity-building project. Since joining IDI, she has provided senior technical support as the Outreach HIV/AIDS Treatment Specialist; technical assistance, capacity building support and program management oversight over several donor-funded programs. IDI Outreach programs are currently implemented in seventeen districts across the country with over 300,000 patients in care in the supported districts.

She has 13 years' experience in the Uganda health sector; over seven of which have been spent implementing large scale Health Systems Strengthening Interventions in resource limited settings, at senior management

Co-investigator: Richard Walwema BMLT, MBA

Mr Walwema is a laboratory scientist with over 30 years of experience in the practice and development of laboratory systems and networks in both the public and private sector (within and outside Uganda). My research interests sit at the interface of Microbiology, HIV, tuberculosis, public health, global health security, total quality management, health system strengthening and capacity building. He has field experience in implementing research and public health programs in low- and middle- income countries in East and Southern Africa (Uganda, Kenya and Swaziland).

He has developed and helped maintain appropriate linkages with national and local government organizations (such as Uganda National Health Laboratory Services (UNHLS), Regional Referral Laboratory and District lab managers, internal and external partners. I have supported the Ministry of Health to develop national policies, strategies and guidelines including the antimicrobial resistance surveillance guidelines directly and through participation in national technical working groups. Outside Uganda, he has with the URC Swaziland to strengthen the laboratory network and with the East African Public Health Laboratory Network to strengthen networking and progress towards international accreditation. He has accumulated vast experience in setting up national systems for implementation of Global Health Security projects with a focus on Antimicrobial resistance, Biosafety and biosecurity, Laboratory system strengthening & emerging infectious disease surveillance.

Co-investigator: Apollo Olowo MPH

Mr Olowo is a public health expert in health services leadership and governance, strategic and operational planning, health systems strengthening and program implementation, monitoring and evaluation. He has 15 years' working in public health and research; seven years of which, managing District health services delivery, and eight years in project performance management, and District capacity building, while employed by Management Sciences for Health, Save the Children International (SCI) and Oxfam respectively. He has worked as team leader for SCI the Ebola Virus Disease outbreak preparedness and response, providing technical support to project staff and district health offices in Rwenzori region, western Uganda. Provided technical oversight of planning and implementation of emergency health project focus on Ebola Virus Disease (EVD) outbreak preparedness in high risk districts in Rwenzori region. Worked with other response partners to support districts develop emergency health plan and contingency plan for EVD preparedness.

He has experience planning as well as leading district entry processes for KAP study using community LQAS surveys in 33 districts in Uganda. Mentored and coached district health team-DHT on use of the combined analytical tools (Lot Quality Assurance Sampling –LQAS, Bottleneck analysis and causal analysis, Continuous Quality Improvement and community Dialogues based on citizen report cards and U reports) to facilitate identification of social services gaps, prioritization of possible solutions and allocation of resources for program improvement to enhance quality of care in the project supported districts.

Co-investigator: Mohamed F. Jalloh, MPH

Mohamed Jalloh is a behavioral epidemiologist at the U.S. Centers for Disease Control and Prevention working on the Demand for Immunization Team. He conducts applied research, designs data-driven interventions, and evaluates innovative approaches to strengthen vaccination demand. Before working in immunization, Mohamed was as an epidemiologist in the Division of Global Health Protection. From 2016-2017 he led and contributed to behavioral and epidemiological assessments to improve mortality surveillance systems. During the 2014-2015 Ebola outbreak in West Africa, he was responsible for the overall management and technical guidance for five national household surveys comprising 100+ data collectors and 25+ supervisors in Sierra Leone and Guinea. In addition, he oversaw the implementation of a digital reporting system in five districts using a network of >2500 community-based reporters sharing alerts on sick people and deaths occurring in communities and providing real-time community feedback regarding response services.

He co-founded FOCUS 1000 in 2012, a non-governmental organization in Sierra Leone. From 2010 to 2012 Mohamed served as a project coordinator in the Program Office of New Connections—a national program of Robert Wood Johnson Foundation providing $100,000 small grants to diverse health researchers in United States. Mohamed holds a bachelor of science in public health from Rutgers University and a master of public health from University of North Carolina–Chapel Hill. He is completing his final year of doctoral studies at Karolinska Institutet with a concentration on Ebola behavioral epidemiology and global

health. Mohamed has authored and contributed to over 30 publications in peer-reviewed journals.

Co-investigator: Vance Brown, MA

Vance Brown (M.A) joined CDC in 2011 with the Office of Public Health Preparedness and Response Office of the Directorate and transitioned to CGH in July 2011 as Public Health Advisor with the Division of Global Disease Detection and Emergency Response (GDDER) was one of the early members of the Global Health Security Branch; Vance served in various capacities for CGH / GDDER and then the Division of Global Health Protection (DGHP) covering a number of GHS Phase 1 & II Countries from 2011 - Present. In July of 2014, Vance joined the Ebola Response efforts in Sierra Leone as the first team lead for Health Promotion; then served as Deputy for the Ebola Response efforts in Nigeria and again on the response in Sierra Leone in 2015 in multiple leadership capacities. In 2016, Vance accepted to serve as Deputy for DGHP Program in Uganda and relocated to country in the summer of 2016. Vance has served as Acting Director for DGHP for the majority of 2017 during staffing transition requiring full range of diplomatic, scientific, and implementation advisement for the USG and Government of Uganda on GHS implementation. Vance has a Bachelor's degree in Political Science and International Relations from Hope College (2005); a Master's degree in International Administration (M.A.), and participated as a member of the Master's International joint program with the United States Peace Corps and University of Denver Josef Korbel School of International Studies (2005 – 2010) where he served as a Health and Economic Development Volunteer in Uganda (2008 – 2010). Vance has language proficiency in Luganda and Spanish.

Co-investigator: Victoria Carter, MPH, PhD

Victoria Carter is the Lead for Risk Communication and Communication Science in CDC's National Center for Emerging and Zoonotic Infectious Diseases' (NCEZID) Health Communication Science Office. She leads communication research activities to inform strategy and messaging, advises on message testing and evaluation efforts, and contributes to risk communication publications and projects, including field deployments for outbreak responses. For the current Ebola outbreak in DRC, she has deployed multiple times to DRC and Uganda as a Risk Communication Technical Advisor, supporting both preparedness and response communication activities.

Victoria served as a communication lead for the STRIVE Ebola vaccine trial during the 2014-2016 West Africa Ebola outbreak. During CDC's Zika response, she worked in CDC's Emergency Operations Center's Joint Information Center and deployed to the field multiple times. Before joining NCEZID, Victoria worked in CDC's National Center for Immunization and Respiratory Diseases on childhood immunization.

Victoria holds a PhD in Mass Communication from the University of Georgia, a Master of Public Health from Dartmouth, and a Bachelor of Arts degree in Communication Studies and Sociology from Clemson University.

Supplement to: Musaazi J, Namageyo-Funa A, Carter VM, et al. Evaluation of community perceptions and prevention practices related to Ebola virus as part of outbreak preparedness in Uganda, 2020. *Glob Health Sci Pract*. 2022;10(3):e2100661. https://doi.org/10.9745/GHSP-D-21-00661

Co-investigator: Rosalind Carter, PhD

Dr. Rosalind Carter is an epidemiologist with the US Centers for Disease Control and Prevention in the Global Immunizations Division. She received her Bachelor's degree in Psychology from Harvard College and a doctorate in Epidemiology from the University of Michigan and completed post-doctoral work at CDC as an Epidemic Intelligence Service Officer assigned to the New York City Department of Health. For more than 20 years, she has conducted research and published on risk factors for maternal-infant HIV transmission and pediatric HIV disease progression and developed expertise in the monitoring and evaluation of HIV programs in the US as well as sub-Saharan Africa as faculty at Columbia University's School of Public Health and UNICEF Headquarters in New York. During the West Africa Ebola outbreak, Dr Carter returned to CDC as the Field Team Lead for the Sierra Leone Trial to Introduce an Ebola Vaccine (STRIVE). In addition to the main trial results, she has co-authored a number of publications describing lessons learned in implementation, cold chain logistics, surveillance and training that have influenced guidelines on the use of unlicensed Ebola vaccines. Her current work includes studying the effective use of vaccines and conduct of research during public health emergencies, and training, planning and implementation of unlicensed Ebola vaccines to protect health care workers in countries at risk for Ebola.

Co-investigator: Rose Apondi, MPH

Rose Apondi, Behavioral Scientist with Centers for Disease Control and Prevention. Rose is currently pursuing a PhD at Antwerp University, holds a Master's in Public Health degree from Liverpool University and bachelor's degree in Social Sciences from Makerere University in Uganda. Rose has written evaluation protocols as investigator and co-author on several scientific manuscripts. Over fifteen years of program design, management, program implementation, program monitoring and evaluation; research experience. Professional knowledge of health and behavioral programming and research; Social and market strategies and research; writing award winning program and research protocols, proposals, designing research studies; developing questionnaires; contracting and managing contracts and grants; supervision of individuals and teams; analyzing quantitative and qualitative data; and writing qualitative and quantitative reports and scientific publications. Key competences in HIV programming and operations research, HIV prevention strategies, Sexual and reproductive health, Gender integration, audits, analysis and programming; translation of evidence-based interventions to programs, training, curriculum development, and policy development; behavioral science expert, design of behavioral interventions for adolescents and adults; monitoring and evaluation of behavioral interventions. Experienced in various research methodologies including quantitative and qualitative research methods, behavioral studies, lifestyle studies, client exit interviews, media surveys; knowledge, attitudes and practices (KAP) surveys; consumer profile, pricing, process monitoring and evaluation (behavioral studies, distribution studies, impact assessment, client satisfaction studies), and formative research such as focus groups, in-depth interviews, participatory rapid appraisal, baseline studies and advertising/packaging/promotional materials development research. First author of scientific manuscripts in peer-reviewed journals. Good knowledge of United States Government contracting procedures for research and program.

Supplement to: Musaazi J, Namageyo-Funa A, Carter VM, et al. Evaluation of community perceptions and prevention practices related to Ebola virus as part of outbreak preparedness in Uganda, 2020. *Glob Health Sci Pract*. 2022;10(3):e2100661.
https://doi.org/10.9745/GHSP-D-21-00661

Annex 3: Interview Consent Script

Assessment of Public Knowledge, Attitudes, and Practices Relating to Ebola Virus Disease (EVD) Prevention and Treatment in Uganda, 2019

Good morning or afternoon. My name is ______________________________. I am working with the Infectious Diseases Institute or IDI, which is part of Makerere University in Kampala. We are working with the Uganda Ministry of Health to protect the health of Ugandans. We are conducting interviews in the community to find out how much people know, what they think and what they do about Ebola. The information we collect will help the Government of Uganda educate people on how to protect themselves and their communities against Ebola.

I am here today to ask if you would like to do this interview. Here is what you should know about participating in this assessment.

- We are interviewing nearly 4000 people in six districts in Uganda, including 32 people in your community.
- The interview will take about 50-60 minutes.
- All of the information you will share will be kept confidential. That means we will not link your name or household to any reports or identify you in any other way.
- Your participation in the interview is voluntary. You may stop the interview at any point. You can refuse to answer any question you do not feel comfortable answering. There will be no penalty or consequences whatsoever for stopping the interview early or not answering any question.
- There are no right or wrong answers. We encourage you to be open and honest in your responses so that the Ministry of Health can get the best information possible.
- We will not pay you for responding but you will receive a bar of soap to thank you for taking the time to talking with us today. We will do our best to answer any question you may have about Ebola or other diseases.
- By participating, you are helping to protect your community and country.

ASK RESPONDENT

1. Do you have any questions about the survey or your potential participation?
   YES → Answer their question(s).
   NO → Continue to next question.

2. Do you want to participate in our interview today?
   YES → Explain that the consent form outlines the information you just gave them and is their written agreement to participate. Ask them to sign it. If needed, review it with them and answer any additional questions.
   NO → End the conversation and respectfully thank them for their time.

Supplement to: Musaazi J, Namageyo-Funa A, Carter VM, et al. Evaluation of community perceptions and prevention practices related to Ebola virus as part of outbreak preparedness in Uganda, 2020. *Glob Health Sci Pract*. 2022;10(3):e2100661. https://doi.org/10.9745/GHSP-D-21-00661

Annex 4: Participant Consent Form

Assessment of Public Knowledge, Attitudes, and Practices Relating to Ebola Virus Disease (EVD) Prevention and Treatment in Uganda, 2019

The Infectious Diseases Institute (IDI), which is part of Makerere University, is working with the Ministry of Health to protect the health of Ugandans. IDI is conducting a community-based assessment on the public's knowledge, attitudes, and practices concerning Ebola virus disease. The information collected will help the Government of Uganda educate people on how to protect themselves and their communities against Ebola.

By choosing to take part in this community-based assessment interview, I understand that:

- People in 6 districts in Uganda are being asked to take part in interviews for the assessment. This includes 16 households in my community.
- The interview will take about 50-60 minutes.
- All of the information I share will be kept confidential. Nothing used in a report will identify me or be linked to my name or household.
- My participation in this assessment is voluntary. I can stop at any time. I can refuse to answer questions I am not comfortable with. There is no penalty if I stop the interview or do not answer any question.
- There are no right or wrong answers. I will strive to be open and honest in my answers so that the best information possible can be collected.
- There is no direct benefit to me for participating, but by answering the questions I am helping to protect my community and country.
- I will receive a bar of soap for taking part in the interview as well as an Ebola prevention and control flyer to help me remember and educate my relatives and community fellows about Ebola.

Supplement to: Musaazi J, Namageyo-Funa A, Carter VM, et al. Evaluation of community perceptions and prevention practices related to Ebola virus as part of outbreak preparedness in Uganda, 2020. *Glob Health Sci Pract*. 2022;10(3):e2100661. https://doi.org/10.9745/GHSP-D-21-00661

By signing this form, I confirm that I have read or have been told, and that I fully understood the above information. I agree to take part in the community-based assessment.

SIGNATURE **or** THUMBPRINT OF PARTICIPANT:

…………………….………………………………………….

DATE (Day, Month, Year): ______/______ /______

If you have any questions, please contact:

Mohammed Lamorde, PhD

Principal Investigator, 2019 Uganda Ebola KAP Survey

Tel: 0772185590

Email: mlamorde@idi.co.ug

Infectious Diseases Institute, McKinnell Knowledge Center, Makerere University, Kampala

You may also contact the Ethics Committee regarding any concerns, injury, or risks posed to you as a result of your participation in the community-based assessment:

Dr. Tom Lutalo

Chairperson, UVRI Research Ethics Committee

Tel: 0701444014 / 0776720539,

Email: tlutalo@uvri.go.ug

Supplement to: Musaazi J, Namageyo-Funa A, Carter VM, et al. Evaluation of community perceptions and prevention practices related to Ebola virus as part of outbreak preparedness in Uganda, 2020. *Glob Health Sci Pract*. 2022;10(3):e2100661. https://doi.org/10.9745/GHSP-D-21-00661

Annex 5: Parental Consent Form

Assessment of Public Knowledge, Attitudes, and Practices Relating to Ebola Virus Disease (EVD) Prevention and Treatment in Uganda, 2019

The Infectious Diseases Institute (IDI), which is part of Makerere University, is working with the Ministry of Health to protect the health of Ugandans. IDI is conducting a community-based assessment on the public's knowledge, attitudes, and practices concerning Ebola virus disease. The information collected will help the Government of Uganda educate people on how to protect themselves and their communities against Ebola.

By letting my child participant in this community-based assessment interview, I understand that:

- People in 6 districts in Uganda are being asked to take part in interviews for the assessment. This includes 16 households in my community.
- The interview will take about 50-60 minutes. We shall record the information from this interview if you grant us your permission.
- All of the information my child shall share will be kept confidential. Nothing used in a report will identify my child or be linked to his/her name or household.
- My child's participation in this assessment is voluntary. He/she can stop at any time. My child can refuse to answer questions she/he is not comfortable with. There is no penalty if my child stops the interview or does not answer any question.
- There are no right or wrong answers. My child will strive to be open and honest in his/her answers so that the best information possible can be collected.
- There is no direct benefit to my child for participating, but by answering the questions he/she is helping to protect my community and country.
- My child will receive a bar of soap for taking part in the interview as well as an Ebola prevention and control flyer to help him/her remember and educate his/her relatives and community fellows about Ebola.

Supplement to: Musaazi J, Namageyo-Funa A, Carter VM, et al. Evaluation of community perceptions and prevention practices related to Ebola virus as part of outbreak preparedness in Uganda, 2020. *Glob Health Sci Pract*. 2022;10(3):e2100661. https://doi.org/10.9745/GHSP-D-21-00661

By signing this form, I confirm that I have read or have been told, and that I fully understood the above information. I am giving permission for my child to take part in the community-based assessment.

NAME OF PARTICIPANT'S PARENT

…………………………………………………………………………….

SIGNATURE **or** THUMBPRINT OF PARTICIPANT'S PARENT:

………………………..……………………………………….

DATE (Day, Month, Year): ______/______ /______

If you have any questions, please contact:

Mohammed Lamorde, PhD

Principal Investigator, 2019 Uganda Ebola KAP Survey

Tel: 0772185590

Email: mlamorde@idi.co.ug

Infectious Diseases Institute, McKinnell Knowledge Center, Makerere University, Kampala

You may also contact the Ethics Committee regarding any concerns, injury, or risks posed to your child as a result of his/her participation in the community-based assessment:

Dr. Tom Lutalo

Chairperson, UVRI Research Ethics Committee

Tel: 0701444014 / 0776720539,

Email: tlutalo@uvri.go.ug

Supplement to: Musaazi J, Namageyo-Funa A, Carter VM, et al. Evaluation of community perceptions and prevention practices related to Ebola virus as part of outbreak preparedness in Uganda, 2020. *Glob Health Sci Pract*. 2022;10(3):e2100661. https://doi.org/10.9745/GHSP-D-21-00661

Annex 6: Questionnaire

Assessment of Public Knowledge, Attitudes, and Practices Relating to Ebola Virus Disease Prevention and Treatment in Uganda, 2019

Data Collection Team Identification

Team ID:	Enumerator ID:	Supervisor ID:
Date:	Time:	Location:

District, Cluster, and Household Identification

**DISTRICT**<br>1. Kasese<br>2. Kisoro<br>3. Arua<br>4. Greater Kampala<br>5. Lamwo<br>6. Busia<br><br>**CLUSTER**<br>Identification number ____ ____ ____<br>Name: ________________________________	**HOUSEHOLD**<br>Identification number ____ ____ ____<br><br>Household size:____ ____<br><br>Respondent category:<br>____ Head of household<br>____ Woman 25 years and above<br>____ Young person ages 15-24 years

***Indicates core items adapted from EVD KAP assessments from West Africa*

**Indicates new items developed specifically for the Uganda EVD preparedness context*

SOCIODEMOGRAPHIC CHARACTERISTICS OF RESPONDENT

1. **Gender**
   a. Male
   b. Female

2. **Age:** ___________ years
   or Year of birth: _________
   (enter 88 if don't know and 99 if declined to respond)

3. **What is your highest level of education attained?** *(single selection; prompted as needed)*
   (any level attended even if not completed)
   a. No formal education
   b. Primary school
   c. Secondary school
   d. Post-secondary and above
   e. Declined to respond

4. **What kind of work do you currently do?** *(single selection; prompted as needed)*
   a. Professional / managerial / technical / assistant professional
   b. Clerical support
   c. Service and sales
   d. Skilled agriculture / forestry / fishery
   e. Craft and related trade
   f. Plant and machine operator / assembler
   g. Elementary occupations
   h. Unemployed
   i. Other (specify)
   j. Declined to respond

5. **What is your religion?** *(single selection; unprompted)*
   a. Catholic
   b. Anglican
   c. Muslim
   d. Pentecostal
   e. Seventh Day Adventist
   f. Other (specify)
   g. Declined to respond

Supplement to: Musaazi J, Namageyo-Funa A, Carter VM, et al. Evaluation of community perceptions and prevention practices related to Ebola virus as part of outbreak preparedness in Uganda, 2020. *Glob Health Sci Pract*. 2022;10(3):e2100661. https://doi.org/10.9745/GHSP-D-21-00661

GENERAL HEALTH

[I will now start by asking you to tell me your opinion about health services in your district]

6. **How confident are you in the health services in your district to treat malaria?****
   (single selection; prompted)
   a. Not at all confident
   b. Somewhat confident
   c. Very confident
   d. Never heard of malaria
   e. Declined to respond

7. **How confident are you in the health services in your district to treat tuberculosis (TB/dry cough)?****
   (single selection; prompted)
   a. Not at all confident
   b. Somewhat confident
   c. Very confident
   d. Never heard of tuberculosis
   e. Declined to respond

8. **How confident are you in the health services in your district to treat Ebola?****
   (single selection; prompted)
   a. Not at all confident
   b. Somewhat confident
   c. Very confident
   d. Never heard of Ebola
   e. Declined to respond

9. **Would you be willing to ride in an ambulance if you had a high fever today?***
   (single selection; prompted)
   a. Yes
   b. Unsure
   c. No
   d. Declined to respond

10. **Whom would you seek care from first if you had a high fever today?***
   (single selection; prompted)
   a. No one from outside the home / home-based care
   b. Traditional healer/spiritual healer
   c. Healthcare worker (e.g. doctor, nurse)
   d. Community health worker (e.g. Village Health Team; VHT)
   e. Pharmacist
   f. Other (specify):______________________________

g. Declined to respond

EBOLA AWARENESS

[I will ask you some questions about Ebola.]

11. Have you heard of any place where people are getting infected with Ebola right now?*
(single selection; prompted)
a. Yes → Go to Q12
b. No → Go to Q13
c. I don't know / not sure → Go to Q13
d. I have never heard of Ebola before this interview → Go to Q13
e. Declined to respond → Go to Q13

12. From what you have heard, where are people getting infected with Ebola right now?*
(multiple selection; do not prompt)
a. None
b. Democratic Republic of Congo
c. Uganda
d. South Sudan
e. Sierra Leone
f. Liberia
g. Guinea
h. No response

13. In which district in Uganda, if any, did some people get infected with Ebola in June 2019?*
(multiple selection; do not read choices)
a. No district in Uganda had cases of Ebola
b. Kisoro
c. Kasese
d. Arua
e. Kampala
f. Lamwo
g. Busia
h. I don't know / not sure
i. Declined to respond

OVERALL EBOLA SENTIMENTS

Interviewer to read to interviewee: I will now ask you to tell me if you agree or disagree with a number of ideas, thoughts or beliefs about Ebola I will read to you. Please remember there is no right or wrong answer; we are only trying to find out what the community thinks and how people feel in order to keep improving Uganda's and Ugandans' performance in preventing and controlling this outbreak.

Supplement to: Musaazi J, Namageyo-Funa A, Carter VM, et al. Evaluation of community perceptions and prevention practices related to Ebola virus as part of outbreak preparedness in Uganda, 2020. *Glob Health Sci Pract*. 2022;10(3):e2100661. https://doi.org/10.9745/GHSP-D-21-00661

14. **Do you agree or disagree that Ebola is a man-made disease?***
    (single selection; prompted)
    a. Agree
    b. Disagree
    c. Don't know / not sure
    d. Declined to respond

15. **Do you agree or disagree that if cases of Ebola start happening in Uganda, your community will fully cooperate with the authorities to stop Ebola from spreading.***
    (single selection; prompted)
    a. Agree
    b. Disagree
    c. Don't know / not sure
    d. Declined to respond

16. **Do you agree or disagree that if cases of Ebola start happening in Uganda, you and your family will fully cooperate with the authorities to stop Ebola from spreading.***
    (single selection; prompted)
    a. Agree
    b. Disagree
    c. Don't know / not sure
    d. Declined to respond

17. **Do you agree or disagree that if cases of Ebola start happening in Uganda, there is nothing someone can do to prevent Ebola from spreading?***
    (single selection; prompted)
    a. Agree
    b. Disagree
    c. Don't know / not sure
    d. Declined to respond

18. **Do you agree or disagree that if cases of Ebola start happening in Uganda, the bodies of people who may die from Ebola will be handled with respect by the authorities?***
    (single selection; prompted)
    a. Agree
    b. Disagree
    c. Don't know / not sure
    d. Declined to respond

Supplement to: Musaazi J, Namageyo-Funa A, Carter VM, et al. Evaluation of community perceptions and prevention practices related to Ebola virus as part of outbreak preparedness in Uganda, 2020. *Glob Health Sci Pract*. 2022;10(3):e2100661.
https://doi.org/10.9745/GHSP-D-21-00661

EBOLA RISK PERCEPTION

[Next I will ask you some questions about how you view your risk of getting Ebola]

19. What level of risk do you think you have in getting Ebola in the next 6 months?
(single selection; prompted)
a. No risk → Go to Q21
b. Small risk → Go to Q20
c. Moderate risk → Go to Q20
d. Great risk → Go to Q20
e. I don't know / not sure → Go to Q22
f. Declined to respond → Go to Q22

20. What is the <u>MAIN</u> reason for which you think you are at risk of getting Ebola?**
(single selection; do not read choices)
a. I have been experiencing signs and symptoms of Ebola
(if so: stop the interview, recommend that the person goes to the nearest health facility, provide the Ebola hotline phone number, record the address)
b. Someone in my family/household/dwelling may have Ebola
(if so: stop the interview, finding out if the person is still in the dwelling, recommend that the person goes to the nearest health facility, provide the Ebola hotline phone number, record the address)
c. I travelled to DRC recently
d. I am a health care professional
e. I live in the same household with a health care professional
f. I eat bush meat /hunt bush meat as my means of livelihood
g. Ebola is everywhere
h. I washed/touched the dead body of someone suspected/confirmed to have had Ebola
i. I have attended a burial/funeral ceremony of someone suspected/confirmed to have had Ebola
j. I may get Ebola from mosquito bites
k. I may get Ebola through the air
l. I have unprotected sex with someone who has survived Ebola
m. Others____
n. I don't know / not sure
o. Declined to respond

21. What is the MAIN reason for which you do not think you are at risk of getting Ebola?**
(single selection; do not read choices)

a. Ebola is only a DRC problem
b. I do not eat or hunt bush meat or bats
c. I am not a health care or medical professional
d. I am a clean person / Ebola only affects unclean people
e. I don't live in an area where there is Ebola
f. I don't come in contact with someone with Ebola
g. God is protecting me
h. I have traditional powers that protect me from Ebola
i. I do not participate in burial ceremonies that involve the handling (touching/washing) of the dead body
j. I avoid all funerals and burials
k. I avoid unprotected contact with bodily fluids
l. I wash my hands with soap or other disinfectants
m. I do not have unprotected sex with someone who has survived Ebola
n. Others____
o. I don't know / not sure/
p. Declined to respond

EBOLA KNOWLEDGE

[Next I will ask you some questions about what causes Ebola, how people get it, ways to prevent it, and what to do if Ebola is suspected]

22. Please tell me what you think are all the possible causes of Ebola?**
(multiple selection; do not read choices)

a. Virus
b. Bats / Monkeys / Chimpanzees / Other wild animals
c. God or higher power
d. Witchcraft
e. Evildoing / Sin
f. Curse
g. Man-made
h. Others ____
i. I don't know/ not sure
j. Declined to respond

23. Please tell me what you think is the ways by which a person can get Ebola?**
(multiple selection; do not read choices)

a. By air
b. Mosquito bite
c. Bad odor or smell
d. Preparing bush meat as a meal (such as chimpanzees, monkeys, and other wild animals)
e. Eating bush meat
f. Eating fruits likely to have been bitten by bats
g. Saliva of an infected person
h. Blood of an infected person
i. Sweat of an infected person
j. Urine of an infected person
k. Feces of an infected person
l. Breast milk of an infected person
m. Sperm or vaginal fluid of an infected person
n. Shaking the hands of an infected person
o. Other physical contact with an infected person
p. God's will
q. Witchcraft
r. Others (specify): ____
s. I don't know / not sure
t. Declined to respond

24. Can you please tell me what are the signs and symptoms of someone infected with Ebola?**
(multiple selection; do not read choices)

a. Any Fever
b. Sudden onset of high fever
c. Severe headache
d. Muscle pain
e. Weakness
f. Diarrhea (with or without blood)
g. Vomiting (with or without blood)
h. Abdominal (stomach) pain
i. Lack of appetite
j. Sore throat
k. Rash
l. Difficulty breathing
m. Bleeding (internal or external)
n. Others (specify):____
o. I don't know / not sure
p. Declined to respond

25. **Do you believe that traditional healers can treat Ebola successfully?****
    (single selection; prompted)
    a. Yes
    b. No
    c. I don't know / not sure
    d. Declined to respond

26. **Do you believe that spiritual healers can treat Ebola successfully?****
    (single selection; prompted)
    a. Yes
    b. No
    c. I don't know / not sure
    d. Declined to respond

27. **Can someone prevent getting Ebola by avoiding mosquito bites?***
    (single selection; prompted)
    a. Yes
    b. No
    c. I don't know / not sure
    d. Declined to respond

28. **Can someone prevent getting Ebola by avoiding funeral or burial rituals that require handling the body of a person who has died from Ebola?****
    (single selection; prompted)
    a. Yes
    b. No
    c. I don't know / not sure
    d. Declined to respond

29. **If a person has Ebola, do you think he/she will have a greater chance of survival if he/she goes immediately to a health facility?****
    (single selection; prompted)
    a. Yes, she will have a greater chance of survival
    b. No, she will not have a greater chance of survival
    c. I don't know / not sure
    d. Declined to respond

30. **If a person with Ebola goes immediately to a health facility, do you think he/she will reduce the chance of spreading it to family/people living with them?****
    (single selection; prompted)
    a. Yes
    b. No

   c. I don't know / not sure
   d. Declined to respond

31. Do you think it is possible to survive and recover from Ebola?**
(single selection; prompted)
   a. Yes
   b. No
   c. I don't know / not sure
   d. Declined to respond

32. Once a man has survived Ebola, do you think he should use a condom during sex?*
(single selection; prompted)
   a. Yes; **for how long? ______________________** **
   b. No
   c. It does not matter
   d. I don't know / not sure
   e. Declined to respond

33. Do you know of any number to call to report a suspected Ebola patient or death?
(single selection; prompted)
   a. Yes, (33b) number:________________
   b. No→ Go to QX
   c. I don't remember/not sure → Go to QX
   d. Declined to respond → Go to QX

PRACTICES & BEHAVIORAL INTENTIONS

[Next, I will ask you some questions to better understand about health issues in your household, how people in the household get care for health problems, and other practices at the household or community level]

34. Has anyone in this household, including you, been sick <u>during the past month</u>?**
(single selection; prompted)
   a. Yes → Go to Q
   b. No → Go to Q
   c. I don't know / not sure →Go to Q
   d. Declined to respond → Go to Q

35. What were the signs and symptoms of the sick household member(s)?**
(multiple selection; do not read choices)
   a. Fever
   b. Headache
   c. Muscle pain
   d. Weakness
   e. Diarrhea (with or without blood)

f. Vomiting (with or without blood)
g. Abdominal (stomach) pain
h. Lack of appetite
i. Sore throat
j. Rash
k. Difficulty breathing
l. Bleeding (internal or external)
m. Chest pain
n. Coughing
o. Others (specify)____
p. Don't remember
q. Declined to respond

36. Did the sick household member(s) go to a hospital or health facility?**
(single selection; prompted)
a. Yes → Go to Q38
b. No → Go to Q37
c. I don't know / not sure→ Go to Q38
d. Declined to respond → Go to Q38

37. What were the reasons for not going to a hospital or health facility?*
(multiple selection; not read choices)
a. Had no money / can't afford to pay
b. Believed the hospital/health facility could not help
c. Preferred to go to a nearby pharmacy instead
d. Preferred to go to a traditional or spiritual healer
e. Other__________________
f. Don't know / not sure
g. No response

38. In the past month, have <u>YOU</u> participated in a funeral/burial ceremony?
(single selection; prompted)
a. Yes → Go to Q39-42
b. No → Go to Q43
c. Declined to respond → Go to Q43

39. If yes, where did the funeral/burial ceremony take place?
(single selection; prompted)
a. In my current district in Uganda
b. Outside of my current district but within Uganda
c. Outside of Uganda (specify country:) ______________________
d. Declined to respond

40. If yes, where did people who attended the funeral/burial come from?*

(Multiple selection; prompted)

a. Everyone came from within my current district in Uganda
b. Some people came from other districts in Uganda
c. Some people came from DRC
d. Other (specify): ______________________________
e. I don't know / remember
f. Declined to answer

41. If yes, what happened at the funeral/burial?*

(multiple selection; do not read choices)

a. Religious leader prayed for the deceased
b. Family members observed burial from a distance
c. Traditional rituals involving physical contact with the corpse were performed
d. Attendees touched each other (hug, shake hands, etc.)
e. None of the above
f. Other (specify): _________________________
g. I don't remember
h. Declined to respond

42. During the funeral / burial ceremony did YOU have any physical contact with the dead body?**

(single selection; prompted)

a. Yes
b. No
c. I don't remember
d. Declined to respond

43. Since you heard of Ebola, have you taken any action to avoid being infected?**

(single selection; prompted)

a. Yes → Go to Q44-45
b. No → Go to Q46
c. I don't know / can't remember → Go to Q46
d. Declined to respond → Go to Q46

44. In what ways have you changed your behavior or taken actions to avoid being infected? **

(multiple selection; do not read choices)

a. I wash my hands with soap and water more often
b. I wash my hands with just water more often
c. I clean my hands with other disinfectants more often
d. I try to avoid crowded places
e. I drink a lot of water / juice
f. I drink traditional herbs

g. I take antibiotics (e.g. penicillin, amoxilin)
h. I wear gloves (if so ask, how many times you change the gloves daily:__________)
i. I try to avoid physical contact with people I suspect may have Ebola
j. I avoid physical contact with everyone
k. I do not participate in burial ceremonies that involve the handling (touching/washing) of the dead body
l. I wash with salt and hot water
m. I use a condom when having sex with someone who has survived Ebola
n. I always use a condom when having sex
o. Others____
p. I don't know / not sure
q. Declined to respond

45. Of the actions you have taken to avoid being infected with Ebola, which one do you think is the most important?*
(multiple selection; do not read choices)
a. I wash my hands with soap and water more often
b. I wash my hands with just water more often
c. I clean my hands with other disinfectants more often
d. I try to avoid crowded places
e. I drink a lot of water / juice
f. I drink traditional herbs
g. I take antibiotics (e.g. penicillin, amoxilin)
h. I wear gloves (if so ask, how many times you change the gloves daily:__________)
i. I try to avoid physical contact with people I suspect may have Ebola
j. I avoid physical contact with everyone
k. I do not participate in burial ceremonies that involve the handling (touching/washing) of the dead body
l. I wash with salt and hot water
m. I use a condom when having sex with someone who has survived Ebola
n. I always use a condom when having sex
o. Others (specify):____
p. I don't know / not sure
q. Declined to respond

46. What would you do if you suspect someone in your family has Ebola?**
(multiple selection; do not read choices)
a. Nothing
b. Report to district heath authorities
c. Help care for the person at home
d. Check the person's temperature by touching their body
e. Avoid all physical contact and bodily fluids of that person

f. Take the person to a health facility
g. Hide the person
h. Others (specify): ____
i. I don't know / not sure
j. Declined to respond

47. What do you think health workers would do if someone suspected of having Ebola goes to the hospital / health facility?**
(multiple selection; do not read choices)
  a. They won't be able to do anything for him/her and may die there
  b. They will take care of him/her
  c. They will definitely cure the person from Ebola
  d. They will find a way to kill the patient
  e. They turn the patient away
  f. Others (specify): ______________________________
  g. I don't know / not sure
  h. Declined to respond

48. How important is it for family members to be able to visit and see a relative who is admitted to a health facility due to Ebola?*
(single selection; prompted)
  a. Important
  b. Somewhat important
  c. Not important
  d. Declined to respond

49. If a family member died, would you accept alternatives to traditional funeral/burial that would NOT involve the touching or washing of the dead body?**
(single selection; prompted)
  a. Yes
  b. No
  c. I don't know / not sure
  d. Declined to respond

EBOLA VACCINATION

[Next I will questions about Ebola vaccines.]

50. Have you heard about an Ebola vaccine <u>before this interview</u>?*
(single selection; prompted)
  a. Yes → Go to Q51-57
  b. No → Go to Q58
  c. I don't know/remember → Go to Q58
  d. Declined to respond → Go to Q58

51. Please say if you agree, somewhat agree, disagree, or have no opinion about the following statement: "If Uganda started having cases of Ebola, an Ebola vaccine is needed to help prevent the spread of the disease in the country"*
(single selection; prompted)
  a. Agree

b. Somewhat agree
c. Disagree
d. I don't know / I'm not sure
e. Declined to respond

52. If there is an Ebola outbreak in your district, who do you think should be the first to get an Ebola vaccine?**
(single selection, do not read choices)
a. Me/my family
b. Healthcare workers
c. Burial teams
d. Political leaders
e. Pregnant women
f. Children
g. Team that is offering the Ebola vaccine
h. People who live in worst affected areas
i. Other: ____
j. No one should get the vaccine
k. I don't know /no opinion
l. Declined to answer

53. If there is an Ebola outbreak in your district, how many people in your community do you think would agree to take an Ebola vaccine if they were offered it?*
(single selection; prompted)
a. No one
b. Some people
c. Most people
d. Everyone
e. I don't know / no opinion
f. Declined to respond

54. If there is an Ebola outbreak in your district, how likely would you be to take an Ebola vaccine for yourself if you were offered it?*
(single selection; prompted)
a. Very likely to take it
b. Somewhat likely to take it
c. Not very likely to take it
d. Not at all likely to take it
e. I don't know / I'm not sure
f. Declined to answer

55. What concerns do you have about taking an Ebola vaccine?*
(multiple selection; do not read choices)
a. I have no concerns about the Ebola vaccine

b. It may cause Ebola
c. It may cause death
d. It may cause side effects like muscle aches and body pain in the short term
e. It may cause infertility or impotence/sexual weakness
f. It cannot prevent Ebola
g. Lack of trust in vaccine manufacturer
h. Lack of trust in the process used to make the vaccine
i. Lack of trust in the team offering the vaccine
j. Lack of trust in the health system
k. Other:________________________
l. I don't know / I'm not sure
m. Declined to respond

56. Have you ever been offered an Ebola vaccine?*
(single selection; prompted)
a. Yes → Go to Q57
b. No → Go to Q58
c. Declined to respond → Go to Q58

57. Did you take the vaccine when it was offered to you?*
(single selection; prompted)
a. Yes
b. No
c. Declined to respond

STIGMA & DISCRIMINATION

58. Would you buy fresh vegetables from a shopkeeper who survived Ebola and has a certificate from a Government Health Facility stating he/she is now Ebola-free?*
(single selection; prompted)
a. Yes
b. No
c. I don't know / not sure
d. Declined to respond

59. Do you think that a school pupil who has survived Ebola and has a certificate from a Government Health Facility stating he/she is Ebola-free puts other pupils in their class at risk of infection?*
(single selection; prompted)
a. Yes
b. No
c. I don't know / not sure

d. Declined to respond

60. Would you welcome someone back into your community/neighborhood after he/she has recovered from Ebola?*
(single selection; prompted)
   a. Yes
   b. No
   c. I don't know / not sure
   d. Declined to respond

INFORMATION SOURCES & EXPOSURE

61. Which Ebola prevention message(s) have you received in the past 6 months?*
(multiple selection; do not read choices)
   a. I haven't received any Ebola messages → Go to Q63
   b. Avoid participating in funeral practices and traditional burials that involve contact with the corpse
   c. Avoid contact with sick people who have Ebola-like symptoms
   d. Report deaths that resemble Ebola to health authorities
   e. Report sick people to health authorities
   f. Wash your hands
   g. Do not eat bush meat
   h. Other (specify): ____
   i. Declined to respond

62. How did you receive the Ebola message?*
(multiple selection; do not read choices)
   a. Radio
   b. Television
   c. Megaphone public announcements
   d. Household visit by a health worker or other Ebola response worker
   e. Church / Mosque / other religious venues
   f. Other community meetings
   g. Film vans
   h. Newspaper /Newsletter/ Other print materials
   i. Posters / flyers
   j. Internet / Blog / Website / Social Media / Facebook
   k. Mobile phone / text messages / WhatsApp
   l. Traditional leaders (chief, village headman, etc.)
   m. Ministry of Health and Sanitation
   n. Informational visits to the school I work at or attend
   o. Other (specify): ____
   p. I don't know / not sure
   q. Declined to respond

63. What topic related to Ebola would you like more information on?
(multiple selection; do not read choices)
a. I don't want any more information on Ebola
b. Ebola outbreak in DRC
c. Sign sand symptoms of Ebola
d. How Ebola is spread
e. How Ebola can be prevented
f. Where people can go to get treatment for Ebola
g. How people who get Ebola can be cared for
h. How people who get Ebola can be safely buried
i. Monitoring of people who come in contact with an Ebola patient
j. Ebola vaccine safety
k. Ebola vaccine duration of protection
l. Ebola vaccine eligibility
m. Ebola survivors transmission of Ebola through casual contact
n. Ebola survivors transmission of Ebola through sexual contact
o. Other (specify): ____________________________________
p. Declined to respond

TRAVEL & VISITATIONS

[We are now almost at the end of the interview. I'd like to ask you about places you have travelled to or have people visit you from.]

64. In the past 6 months, did you travel to a place outside your district?**
(single selection; prompted)
a. Yes → Go to Q65
b. No → Go to Q66
c. Declined to respond → Go to Q66

65. Where did you travel to?**
(multiple selection; do not read choices)
a. Within Uganda (specify district):__________*(use dropdown list in tablet)*
b. Democratic Republic of Congo (DRC)
c. South Sudan (specify):___________________ *(use dropdown list in tablet)*
d. Kenya (specify):_________________________ *(use dropdown list in tablet)*
e. Rwanda (specify):_______________________ *(use dropdown list in tablet)*
f. Kenya (specify):_________________________ *(use dropdown list in tablet)*
g. Other (specify): _________________________
h. Declined to respond

66. **In the past 6 months, did someone from a place outside your district traveled to visit your or another household member****
    (single selection; prompted)
    a. Yes → Go to Q67
    b. No → Go to CLOSING script
    c. I don't know / not sure → Go to Q68
    d. Declined to respond → Go to Q68

67. **Where did the person(s) traveled from?****
    (multiple selection; do not read choices)
    a. Within Uganda (specify district):__________ *(use dropdown list in tablet)*
    b. Democratic Republic of Congo (DRC)
    c. South Sudan (specify):__________________ *(use dropdown list in tablet)*
    d. Kenya (specify):_________________________ *(use dropdown list in tablet)*
    e. Rwanda (specify):_______________________ *(use dropdown list in tablet)*
    f. Kenya (specify):_________________________ *(use dropdown list in tablet)*
    g. Other (specify): _________________________*(use dropdown list in tablet)*
    h. Declined to respond

INVESTIGATION THERAPEUTICS

68. ***Imagine your relative had Ebola, and there was a medical treatment that has shown to be safe when used in humans but it is unknown if the treatment actually works.***
    Would you be willing to accept this treatment for your relative?*
    (single selection; prompted)
    a. Yes
    b. No
    c. I don't know / not sure
    d. Declined to respond

Supplement to: Musaazi J, Namageyo-Funa A, Carter VM, et al. Evaluation of community perceptions and prevention practices related to Ebola virus as part of outbreak preparedness in Uganda, 2020. *Glob Health Sci Pract*. 2022;10(3):e2100661. https://doi.org/10.9745/GHSP-D-21-00661

CLOSING SCRIPT

- *Thank you for taking the time to discuss these important issues with me.*
- *Again, please rest assured that your responses will be kept confidential.*
- *Your name or any other contact of your family or household will not be included in the report.*
- *The responses you have provided will help in improving risk communication activities and other efforts aimed at protecting the people of Uganda against Ebola.*
- *Once again, thank you very much.*

DO YOU HAVE ANY QUESTIONS FOR ME?

Instructions to Interview: Answer questions you are have been trained to answer, then inform participant that he/she can call the supervisor (information below) if they have additional questions about participating or they should consult the number on the Ebola flyer left behind if they have questions about Ebola.

Supervisor Name:_______________________________________

Phone number: ____

References

1. CDC. Ebola virus disease: transmission. 2018. https://www.cdc.gov/vhf/ebola/transmission/index.html (accessed September 2018).

2. CDC. Ebola: signs and symptoms. 2018. https://www.cdc.gov/vhf/ebola/symptoms/index.html (accessed September 2018).

3. WHO. Ebola Situation Report. 2019. https://afro.who.int/publications/ebola-virus-disease-outbreak-uganda-situation-reports (accessed August 1 2019).

4. Funk S, Ciglenecki I, Tiffany A, et al. The impact of control strategies and behavioural changes on the elimination of Ebola from Lofa County, Liberia. *Philos Trans R Soc Lond B Biol Sci* 2017; 372(1721).

5. Barbarossa MV, Denes A, Kiss G, Nakata Y, Rost G, Vizi Z. Transmission Dynamics and Final Epidemic Size of Ebola Virus Disease Outbreaks with Varying Interventions. *PLoS One* 2015; 10(7): e0131398.

6. Fast SM, Mekaru S, Brownstein JS, Postlethwaite TA, Markuzon N. The Role of Social Mobilization in Controlling Ebola Virus in Lofa County, Liberia. *PLoS Curr* 2015; 7.

7. Gillespie AM, Obregon R, El Asawi R, et al. Social Mobilization and Community Engagement Central to the Ebola Response in West Africa: Lessons for Future Public Health Emergencies. *Glob Health Sci Pract* 2016; 4(4): 626-46.

8. Lee-Kwan SH, DeLuca N, Bunnell R, Clayton HB, Turay AS, Mansaray Y. Facilitators and Barriers to Community Acceptance of Safe, Dignified Medical Burials in the Context of an Ebola Epidemic, Sierra Leone, 2014. *J Health Commun* 2017; 22(sup1): 24-30.

9. Richards P, Amara J, Ferme MC, et al. Social pathways for Ebola virus disease in rural Sierra Leone, and some implications for containment. *PLoS neglected tropical diseases* 2015; 9(4): e0003567.

10. Tiffany A, Dalziel BD, Kagume Njenge H, et al. Estimating the number of secondary Ebola cases resulting from an unsafe burial and risk factors for transmission during the West Africa Ebola epidemic. *PLoS neglected tropical diseases* 2017; 11(6): e0005491.

11. Camacho A, Kucharski A, Aki-Sawyerr Y, et al. Temporal Changes in Ebola Transmission in Sierra Leone and Implications for Control Requirements: Real-time Modelling Study. *PLoS Curr* 2015; 7.

12. Fallah M, Dahn B, Nyenswah TG, et al. Interrupting Ebola Transmission in Liberia Through Community-Based Initiatives. *Ann Intern Med* 2016; 164(5): 367-9.

13. Jalloh MF, Robinson SJ, Corker J, et al. Knowledge, Attitudes, and Practices Related to Ebola Virus Disease at the End of a National Epidemic - Guinea, August 2015. *MMWR Morb Mortal Wkly Rep* 2017; 66(41): 1109-15.

14. Jalloh MF, Sengeh P, Monasch R, et al. National survey of Ebola-related knowledge, attitudes and practices before the outbreak peak in Sierra Leone: August 2014. *BMJ Glob Health* 2017; 2(4): e000285.

15. Li W, Jalloh MF, Bunnell R, et al. Public Confidence in the Health Care System 1 Year After the Start of the Ebola Virus Disease Outbreak - Sierra Leone, July 2015. *MMWR Morb Mortal Wkly Rep* 2016; 65(21): 538-42.

16. Jalloh MF, Bunnell R, Robinson S, et al. Assessments of Ebola knowledge, attitudes and practices in Forecariah, Guinea and Kambia, Sierra Leone, July-August 2015. *Philos Trans R Soc Lond B Biol Sci* 2017; 372(1721).

17. WHO. Ebola outbreak in the Democratic Republic of the Congo declared a Public Health Emergency of International Concern. 2019. https://www.who.int/news-room/detail/17-07-2019-ebola-outbreak-in-the-democratic-republic-of-the-congo-declared-a-public-health-emergency-of-international-concern (accessed July 2019).

18. Vinck P, Pham PN, Bindu KK, Bedford J, Nilles EJ. Institutional trust and misinformation in the response to the 2018-19 Ebola outbreak in North Kivu, DR Congo: a population-based survey. *Lancet Infect Dis* 2019; 19(5): 529-36.

19. Bedrosian SR, Young CE, Smith LA, et al. Lessons of Risk Communication and Health Promotion - West Africa and United States. *MMWR Suppl* 2016; 65(3): 68-74.

20. Bedson J, Jalloh MF, Pedi D, et al. Community Engagement during outbreak response: standards, approaches, and lessons from the 2014-2016 Ebola outbreak in Sierra Leone. 2019: 661959. https://www.biorxiv.org/content/10.1101/661959v1

21. Fitzmaurice AG, Mahar M, Moriarty LF, et al. Contributions of the US Centers for Disease Control and Prevention in Implementing the Global Health Security Agenda in 17 Partner Countries. *Emerg Infect Dis* 2017; 23(13).
