# Supplementary material for: Evaluation of Community Perceptions and Prevention Practices Related to Ebola Virus as Part of Outbreak Preparedness in Uganda, 2020
Source: Glob Health Sci Pract. 2022 Jun 29;10(3):e2100661. doi: 10.9745/GHSP-D-21-00661 (PMC9242603; doi:10.9745/GHSP-D-21-00661)
Supplement: GHSP-D-21-00661-supplement-3.pdf [file GHSP-D-21-00661-supplement-3.pdf]

Supplement to: Musaazi J, Namageyo-Funa A, Carter VM, et al. Evaluation of community perceptions and prevention practices related to Ebola virus as part of outbreak preparedness in Uganda, 2020. *Glob Health Sci Pract*. 2022;10(3):e2100661. https://doi.org/10.9745/GHSP-D-21-00661

Supplement 3. Detailed Summary Tables Across the Six Districts

Table S1: Characteristics of survey respondents across the six districts (unweighted)

Characteristics *	Overall	Low-risk districts		High-risk districts			
	N=3485<br>n (%)	Busia<br>N=634<br>n (%)	Lamwo<br>N=593<br>n (%)	Arua<br>N=506<br>n (%)	Kampala<br>N=511<br>n (%)	Kisoro<br>N=612<br>n (%)	Kasese<br>N=629<br>n (%)
**Respondent category**							
Household head	1769 (50.8)	318 (50.2)	298 (50.3)	262 (51.8)	262 (51.3)	307 (50.2)	322 (51.2)
Woman aged ≥25 years	1019 (29.2)	177 (27.9)	169 (28.5)	138 (27.3)	160 (31.3)	177 (28.9)	198 (31.5)
Young person aged 15-24 years	697 (20.0)	139 (21.9)	126 (21.3)	106 (20.9)	89 (17.4)	128 (20.9)	109 (17.3)
**Age (years)** ♀							
15 – 24	761 (21.8)	144 (22.7)	140 (23.6)	129 (25.5)	102 (19.9)	134 (21.9)	112 (17.8)
25 – 34	878 (25.2)	149 (23.5)	137 (23.1)	158 (31.2)	173 (33.9)	128 (20.9)	133 (21.1)
35 – 44	715 (20.5)	126 (19.9)	118 (19.9)	78 (15.4)	115 (22.5)	132 (21.6)	146 (23.2)
45 – 59	686 (19.7)	127 (20.0)	115 (19.4)	85 (16.8)	79 (15.5)	108 (17.6)	172 (27.4)
60 and above	445 (12.8)	88 (13.9)	83 (14.0)	56 (11.1)	42 (8.2)	110 (18.0)	66 (10.5)
**Sex**							
Female	2106 (60.4)	349 (55.1)	344 (58.0)	300 (59.3)	392 (76.7)	376 (61.4)	345 (54.8)
Male	1379 (39.6)	285 (44.9)	249 (42.0	206 (40.7)	119 (23.3)	236 (38.6)	284 (45.2)
**Education level** ¥							
No formal education	640 (18.4)	77 (12.2)	124 (21.0)	85 (16.8)	28 (5.5)	204 (33.4)	122 (19.4)
Primary	1747 (50.2)	339 (53.6)	311 (52.5)	317 (62.6)	164 (32.2)	324 (53.0)	292 (46.4)
Secondary and higher	1093 (31.4)	216 (34.2)	157 (26.5)	104 (20.6)	318 (62.3)	83 (13.6)	215 (34.2)
**Occupation** ‡							
Agriculture	1504 (43.2)	272 (43.0)	298 (50.5)	164 (32.5)	27 (5.3)	398 (65.0)	345 (54.8)
Service and sales	572 (16.4)	116 (18.4)	49 (8.3)	86 (17.0)	153 (29.9)	42 (6.9)	126 (20.0)
Elementary occupations	253 (7.3)	38 (6.0)	12 (2.0)	132 (26.1)	51 (10.0)	7 (1.1)	13 (2.1)
Professional job	201 (5.8)	28 (4.4)	19 (3.2)	14 (2.8)	85 (16.6)	14 (2.3)	41 (6.5)
Casual labour	24 (0.7)	2 (0.3)	4 (0.7)	5 (1.0)	8 (1.6)	2 (0.3)	3 (0.5)
Unemployed †	925 (26.6)	176 (27.9)	208 (35.3)	104 (20.6)	187 (36.6)	149 (24.4)	101 (16.1)

Response rate: Overall 91%, Low-risk districts 92%, High-risk districts 89%
♀ Age categorized as per the 2016 Uganda Demographic and Health Survey.
¥ Missing values: Education (n=5, <1%), Occupation (n=6, <1%), Religion (n=2, <1%)
‡ Occupation categories derived from the 2016 Uganda Demographic and Health Survey
† Unemployed mostly included students = 97 (10.5%)
§ Christians include grouped response categories for Anglicans, Catholics, Pentecostals and Seventh day Adventists
* Except from education level characteristic (as indicated), all other characteristics had no missing data.
§ Christians include: Anglicans, Catholics, Pentecostals and Seventh day Adventists

Supplement to: Musaazi J, Namageyo-Funa A, Carter VM, et al. Evaluation of community perceptions and prevention practices related to Ebola virus as part of outbreak preparedness in Uganda, 2020. *Glob Health Sci Pract*. 2022;10(3):e2100661. https://doi.org/10.9745/GHSP-D-21-00661

Table S2. Exposure to Ebola-related messages and perceptions of information gap—Uganda, 2020

	**All districts N = 3,485** % (95% CI)	**Low-risk districts**		**High-risk districts**			
		Busia N=634 n (%)	Lamwo N=593 n (%)	Arua N=506 n (%)	Kampala N=511 n (%)	Kisoro N=612 n (%)	Kasese N=629 n (%)
**Awareness of EVD**							
Aware of EVD outbreak in DRC	47.1 (43.2, 51.0)	41.3 (33.8, 49.3)	29.8 (24.2, 36.1)	62.4 (52.5,71.4)	32.4 (25.9,39.6)	90.6 (80.4,95.7)	75.5 (68.3,81.5)
**Perceived risk of contracting EVD** ¥ €							
No risk	48.6 (44.2, 52.9)	43.4 (36.6,50.5)	52.2 (44.8,59.5)	76.7 (65.7,85.0)	45.4 (37.5,53.4)	44.9 (37.9,52.0)	39.6 (34.5,45.0)
Small risk	23.2 (20.9, 25.7)	23.4 (18.3,29.3)	35.1 (28.1,42.7)	11.7 (7.2,18.3)	27.2 (23.3,31.5)	23.9 (18.5,30.4)	14.1 (10.2,19.2)
Moderate risk	8.9 (7.6, 10.5)	24.1 (18.4,30.9)	9.3 (5.3,15.9)	4.8 (2.8,8.3)	5.5 (4.0,7.6)	9.1 (6.1,13.5)	12.8 (8.6,18.8)
High risk	19.3 (16.4, 22.6)	9.1 (5.7,14.2)	3.4 (1.5,7.4)	6.8 (3.6,12.4)	21.9 (16.7,28.3)	22.1 (12.8,35.2)	33.4 (29.2,37.9)
**Ebola messages received in past 6 months** ¥							
Did not receive any Ebola messages	50.0 (48.0, 51.9)	70.6 (64.1,76.4)	71.0 (63.9,77.2)	26.4 (20.9,32.7)	65.5 (62.4,68.5)	10.7 (8.0,14.2)	3.7 (1.8,7.4)
Hand washing	34.0 (31.0, 37.2)	17.3 (11.7,24.8)	10.7 (7.5,15.1)	45.6 (37.4,54.1)	19.5 (14.9,25.2)	67.5 (62.9,71.7)	80.3 (72.2,86.6)
Avoid physical contact with people	31.7 (28.5, 35.1)	12.2 (8.7,16.8)	19.7 (15.2,25.1)	37.8 (30.7,45.4)	28.1 (22.7,34.3)	51.1 (44.0,58.1)	49.3 (41.9,56.6)
Avoid participating in funeral practices and traditional burials that involve contact with the corpse	18.3 (16.0, 20.8)	4.7 (3.1,7.0)	13.3 (9.1,19.0)	25.7 (20.5,31.7)	17.7 (14.0,22.1)	34.9 (24.4,47.1)	18.1 (13.3,24.1)
Report sick people to health authorities	15.6 (13.8, 17.6)	9.2 (5.8,14.4)	7.7 (4.9,11.7)	12.5 (8.9,17.2)	14.1 (11.2,17.6)	29.0 (24.8,33.7)	24.9 (20.7,29.6)
Report deaths that resemble Ebola to health authorities	13.9 (12.2, 15.7)	3.0 (1.7,5.2)	11.7 (7.7,17.5)	16.9 (11.0,25.1)	13.7 (11.1,16.8)	17.7 (14.3,21.6)	19.0 (16.0,22.3)
Avoid eating bush meat	12.8 (10.4, 15.7)	0.8 (0.3,2.1)	0.3 (0.1,1.0)	12.8 (9.0,18.1)	9.4 (5.4,15.8)	20.3 (15.8,25.7)	35.0 (30.5,39.7)
Avoid overcrowded places	1.0 (0.6, 1.6)	0	0.2 (<0.1,1.3)	3.8 (2.0,7.2)	0.4 (0.1,1.7)	0.5 (0.2,1.5)	2.0 (1.0,4.0)
**Reported source of EVD information**¥₺							
Radio	65.2 (60.8, 69.3)	63.2 (48.7,75.6)	80.1 (69.3,87.8)	74.1 (64.9,81.6)	55.3 (48.0,62.3)	77.6 (66.2,85.9)	74.6 (68.3,80.1)
Television	35.3 (30.8, 40.1)	16.5 (8.1,30.8)	0.7 (0.2,2.9)	3.7 (1.9,6.8)	63.1 (54.1,71.3)	3.0 (1.8,5.2)	7.0 (5.0,9.7)
Church or mosque	27.5 (20.2,36.2)	4.6 (1.8,11.6)	10.5 (5.2,20.1)	28.7 (18.4,41.8)	25.9 (13.4,44.1)	25.9 (21.6,30.8)	53.2 (47.6,58.7)
Community meetings	26.3 (20.6, 32.9)	18.1 (11.7,26.9)	21.4 (15.4,28.9)	26.3 (16.7,38.8)	22.6 (13.0,36.3)	35.2 (27.7,43.6)	39.7 (34.9,44.6)
Household visits	20.0 (10.9, 34.0)	5.5 (2.8,10.6)	9.4 (4.1,20.1)	13.2 (9.6,17.9)	29.7 (12.3,56.0)	11.7 (5.8,22.5)	9.9 (6.2,15.6)
Posters or flyers	10.1 (7.1, 14.3)	0.3 (<0.1,1.8)	3.8 (1.4,9.8)	16.2 (11.2,23.0)	12.5 (7.1,21.1)	11.8 (9.0,15.3)	4.7 (2.9,7.5)
Newspapers	8.3 (6.1, 11.3)	6.0 (2.5,13.6)	0.6 (0.1,2.7)	6.3 (3.6,10.8)	12.4 (8.3,18.3)	2.1 (1.2,3.6)	2.9 (1.6,5.0)
Megaphone public announcements	8.0 (3.3, 18.0)	1.3 (0.3,5.1)	2.4 (0.6,9.1)	1.8 (0.7,4.3)	13.8 (4.9,33.3)	0.3 (0.1,1.6)	3.3 (1.6,6.7)
Others:							
Film vans	4.6 (1.4,14.5)	0	0	0.6 (0.1,2.8)	8.5 (2.2,27.7)	2.3 (0.9,5.8)	0.9 (0.3,2.8)
Internet or blog or social media	4.5 (3.0,6.8)	0.3 (<0.1,1.9)	0.4 (0.1,3.0)	1.2 (0.3,4.7)	8.3 (5.3,12.8)	0.5 (0.1,2.3)	1.1 (0.4,2.6)
Mobile phone (text messages or whatsup)	8.9 (4.1,18.4)	3.7 (1.6,8.5)	2.4 (0.6,9.1)	2.2 (1.0,4.6)	14.0 (5.0,33.3)	1.2 (0.5,2.9)	7.3 (4.8,11.0)
Traditional leaders	5.2 (2.0,13.0)	0.3 (<0.1,1.9)	7.4 (4.0,13.1)	1.6 (0.6,4.2)	7.2 (1.8,25.1)	4.7 (2.8,7.7)	4.6 (1.9,10.6)
Ministry of health	2.3 (1.1,4.7)	1.3 (0.4,4.0)	11.1 (6.7,17.9)	11.9 (8.3,16.6)	12.4 (7.3,20.5)	10.0 (7.1,13.9)	2.3 (1.1,4.7)
At health facility	0.9 (0.5,1.7)	0.1 (<0.1,0.8)	0	1.6 (0.6,4.6)	0.5 (0.1,3.1)	4.8 (2.5,8.9)	1.0 (0.4,2.1)
From school	5.4 (3.5,8.2)	3.3 (1.5,6.8)	5.8 (2.5,12.7)	4.9 (2.8,8.3)	6.0 (2.8,12.4)	6.8 (4.5,10.0)	4.3 (2.9,6.4)

Supplement to: Musaazi J, Namageyo-Funa A, Carter VM, et al. Evaluation of community perceptions and prevention practices related to Ebola virus as part of outbreak preparedness in Uganda, 2020. *Glob Health Sci Pract*. 2022;10(3):e2100661. https://doi.org/10.9745/GHSP-D-21-00661

	**All districts** **N = 3,485** % (95% CI)	**Low-risk districts**		**High-risk districts**			
		Busia N=634 n (%)	Lamwo N=593 n (%)	Arua N=506 n (%)	Kampala N=511 n (%)	Kisoro N=612 n (%)	Kasese N=629 n (%)
**Reported EVD information gap ‡ ¥**							
How EVD is prevented	64.1 (58.6, 69.3)	69.3 (62.7,75.3)	77.5 (70.3,83.4)	52.9 (41.7,63.7)	60.1 (52.7,67.0)	74.2 (61.1,84.0)	73.5 (43.7,90.9)
How EVD is spread	45.8 (40.1, 51.6)	59.4 (50.7,67.6)	63.9 (56.1,71.1)	19.4 (12.9,28.3)	40.9 (34.3,47.8)	68.1 (53.4,79.9)	57.3 (28.2,82.1)
Signs and symptoms of EVD	40.8 (34.3, 47.7)	61.5 (53.6,68.8)	56.9 (50.2,63.4)	17.7 (9.5,30.7)	37.8 (29.1,47.3)	61.0 (47.9,72.7)	40.0 (15.3,71.1)
Where to go for EVD treatment	29.8 (24.6, 35.7)	36.4 (25.0,49.6)	30.3 (25.9,35.1)	14.9 (8.2,25.6)	32.4 (26.0,39.5)	17.2 (6.8,37.1)	34.4 (13.7,63.4)
How to care for an EVD patient	20.1 (15.1, 26.2)	33.4 (24.0,44.2)	21.8 (17.6,26.8)	11.6 (6.5,19.8)	20.8 (13.1,31.4)	8.4 (2.6,23.5)	19.8 (6.8,45.6)
Others:							
Need information on EVD outbreak in DRC	3.6 (1.9,6.6)	2.0 (0.8,4.5)	0.9 (0.2,3.8)	4.0 (1.7,9.3)	2.3 (0.9,5.5)	12.7 (4.9,29.0)	5.8 (0.6,36.3)
Safe burial of person died of EVD	8.1 (5.4,12.0)	28.2 (18.6,40.4)	12.5 (8.3,18.3)	5.8 (3.0,11.2)	1.7 (0.5,5.7)	1.9 (0.4,8.2)	18.8 (6.3,44.6)
Monitoring contacts with an EVD patient	6.5 (4.1,10.0)	26.3 (16.6,39.0)	4.7 (2.7,8.1)	1.1 (0.2,8.2)	1.3 (0.5,3.9)	0.8 (0.1,6.5)	17.5 (6.0,41.2)
EVD vaccine safety	13.2 (9.1,18.9)	28.4 (19.2,39.9)	4.9 (2.5,9.2)	5.8 (2.9,11.3)	9.0 (4.7,16.6)	5.8 (2.3,14.0)	29.8 (11.2,58.9)
EVD vaccine duration of protection	4.7 (2.9,7.4)	25.6 (15.8,38.6)	2.5 (1.2,5.2)	2.2 (0.8,5.7)	0.9 (0.3,3.4)	4.6 (1.3,14.9)	5.2 (0.6,33.7)
EVD vaccine eligibility	5.9 (4.1,8.3)	28.5 (19.7,39.2)	1.5 (0.5,4.7)	0	3.0 (1.9,4.8)	4.1 (1.2,13.7)	6.5 (1.1,29.5)
EVD survivors' transmission through casual contact	3.2 (2.1,4.9)	25.4 (15.2,39.1)	1.5 (0.6,3.4)	2.1 (0.5,8.3)	0.3 (0.1,1.7)	0	0
EVD survivors' transmission through sexual contact	4.0 (2.3,6.8)	24.1 (14.3,37.8)	1.6 (0.6,4.3)	1.1 (0.2,8.2)	0	0	8.0 (1.7,30.7)

EVD: Ebola virus disease; DRC: Democratic Republic of Congo

¶ Design based F-statistic p values for comparing high risk versus low risk districts.

¥ Missing values: Perceived risk of contracting EVD (n=51, 1%), preventive Ebola messages received (n=113, 3%), source of EVD information (n=44, 2%), EVD information gap (n= 2,130, 61%)

€ Don't know responses excluded from the risk perception item (n=828, 24%)

₺ Denominator was number of participants who reported ever received preventive EVD messages

Supplement to: Musaazi J, Namageyo-Funa A, Carter VM, et al. Evaluation of community perceptions and prevention practices related to Ebola virus as part of outbreak preparedness in Uganda, 2020. *Glob Health Sci Pract*. 2022;10(3):e2100661. https://doi.org/10.9745/GHSP-D-21-00661

Table S3. Ebola-related knowledge, attitudes, intentions, and self-reported behaviors—Uganda, 2020

	All districts N = 3,485 % (95%CI)	Low-risk districts		High-risk districts			
		Busia N=634 n (%)	Lamwo N=593 n (%)	Arua N=506 n (%)	Kampala N=511 n (%)	Kisoro N=612 n (%)	Kasese N=629 n (%)
**Knowledge of key EVD signs /symptoms ¥**							
Vomiting	51.0 (47.9, 54.1)	56.2 (49.0,63.3)	60.1 (52.8,67.0)	47.0 (37.0,57.0)	53.2 (48.6,57.7)	48.6 (40.0,57.2)	40.5 (31.5,50.2)
Diarrhea	50.4 (45.0, 55.7)	53.4 (46.2,60.5)	44.6 (36.0,53.7)	43.7 (32.1,56.0)	55.9 (45.9,65.4)	46.4 (37.9,55.1)	38.9 (32.3,46.0)
Fever	43.6 (40.3, 46.9)	28.3 (21.7,35.8)	34.5 (29.0,40.5)	46.4 (37.6,55.6)	43.1 (37.4,49.0)	45.0 (38.6,51.5)	56.9 (51.8,61.8)
Severe headache	21.5 (19.5, 23.6)	19.6 (14.1,26.6)	29.1 (24.1,34.6)	42.9 (34.5,51.6)	11.6 (9.4,14.3)	30.5 (24.8,36.8)	31.6 (25.6,38.3)
Knowledge of all four above signs/symptoms	4.8 (3.4, 6.7)	*3.4 (1.4,8.2)*	*7.8 5.1,11.8)*	*14.9 (7.6,27.3)*	*1.3 (0.3,4.6)*	*6.8 (4.6,10.0)*	*7.1 (3.8,12.9)*
Others symptoms:							
Bleeding (internal/external)	46.9 (44.8,48.9)	31.4 (24.7,39.0)	50.7 (45.5,56.0)	65.3 (59.8,70.4)	32.8 (30.0,35.7)	78.8 (75.3,82.0)	74.7 (66.5,81.4)
Weakness	13.0 (10.7,15.5)	10.9 (7.9,14.9)	14.9 (11.9,18.4)	14.0 (10.5,18.4)	12.6 (8.8,17.6)	13.2 (10.3,16.9)	13.8 (10.2,18.4)
Abdominal pain	6.4 (4.3,9.5)	7.3 (4.8,11.1)	7.9 (5.6,10.9)	10.3 (5.9,17.3)	4.0 (1.2,12.6)	11.8 (9.1,15.1)	7.7 (5.9,9.9)
Muscle pain	4.8 (3.8,6.1)	4.6 (2.4, 8.5)	5.3 (3.8,7.3)	4.1 (1.7,10.0)	3.5 (2.1,5.6)	9.1 (6.5,12.7)	8.0 (5.1,12.5)
Sore throat	2.9 (2.2,4.0)	2.2 (1.2,3.9)	2.7 (1.5,4.8)	2.5 (1.0,6.4)	3.2 (2.0,5.1)	2.0 (0.6,6.2)	3.4 (2.0,5.8)
Rash	2.8 (1.7,4.6)	1.8 (1.0,3.1)	2.1 (1.1,4.0)	4.5 (2.5,7.9)	2.9 (1.1,7.1)	1.4 (0.8,2.6)	2.6 (1.1,5.8)
Lack of appetite	2.7 (2.1,3.4)	2.3 (1.2,4.5)	6.1 (4.2,8.8)	4.8 (2.8,8.3)	1.4 (0.8,2.4)	3.6 (2.2,5.7)	3.6 (2.3,5.8)
Difficulty breathing	2.2 (1.5,3.1)	4.0 (2.4,6.5)	2.5 (1.3,4.9)	2.0 (0.9,4.4)	1.1 (0.4,3.4)	1.1 (0.5,2.3)	5.0 (3.5,7.0)
**Knowledge of EVD prevention and treatment**							
Early medical care increases chance of survival ¥	85.4 (83.3, 87.2)	80.6 (76.7,83.9)	79.6 (74.4,83.9)	84.3 (81.7,86.7)	86.4 (82.6,89.5)	78.3 (74.4,81.8)	91.9 (85.5,95.6)
Early medical care reduces household transmission ¥	82.2 (79.3, 84.9)	81.1 (76.4,85.1)	73.8 (69.3,77.9)	79.7 (74.8,83.8)	81.6 (75.8,86.3)	87.1 (83.2,90.2)	88.6 (82.2,92.9)
Preventable by avoiding contact with infected corpse¥	76.8 (74.0, 79.4)	65.8 (57.7,73.1)	73.9 (68.6,78.6)	71.6 (65.8,76.8)	79.0 (73.9,83.3)	85.8 (81.5,89.3)	78.7 (74.7,82.1)
Knowledge of all three EVD knowledge measures ¥	63.0 (60.1, 65.8)	55.1 (47.1,62.9)	53.8 (48.5,59.0)	57.4 (49.0,65.3)	64.8 (60.1,69.3)	67.9 (62.8,72.6)	68.7 (61.8,74.8)
**Misconceptions of EVD**							
EVD is transmissible by air ¥	22.2 (19.6, 25.0)	42.5 (32.4,53.3)	42.9 (38.4,47.6)	21.7 (16.0,28.7)	21.7 (17.6,26.5)	12.4 (7.8,19.1)	5.6 (3.4,9.0)
Spiritual healers can successfully treat EVD ¥	9.7 (7.9, 11.8)	7.5 (4.6,11.8)	8.2 (5.4,12.1)	4.5 (2.8,7.3)	13.2 (10.0,17.2)	2.8 (1.8,4.3)	7.5 (4.6,12.0)
Traditional healers can successfully treat EVD ¥	2.9 (2.3, 3.7)	6.0 (3.4,10.2)	2.5 (1.3,4.5)	2.6 (1.4,4.7)	2.3 (1.5,3.4)	1.9 (1.0,3.6)	3.9 (2.2,6.7)
Rejection of all three EVD misconception measures	70.4 (67.6, 73.0)	60.0 (51.7,67.7)	50.3 (45.4,55.3)	69.7 (63.4,75.3)	71.7 (66.8,76.2)	77.0 (70.3,82.6)	78.9 (74.2,82.9)
**Comprehensive knowledge**	45.4 (41.8,49.2)	34.2 (25.7,43.8)	28.4 (22.9,34.5)	40.7 (31.3,50.9)	46.6 (40.3,53.0)	57.1 (51.5,62.6)	55.1 (48.8,61.3)
**Attitudes toward EVD survivors**							
Would welcome back survivor into the community ¥	61.8 (58.4, 65.2)	50.2 (42.8,57.5)	70.3 (65.2,75.0)	72.9 (62.9,81.0)	59.6 (53.8,65.3)	47.2 (40.8,53.7)	72.0 (65.3,77.8)
Ebola survivor student does not put class at risk of EVD ¥	56.4 (52.0, 60.6)	50.8 (44.7,56.8)	39.6 (34.2,45.4)	56.3 (50.9,61.5)	61.7 (53.3,69.6)	53.2 (46.1,60.2)	50.3 (44.9,55.7)

Supplement to: Musaazi J, Namageyo-Funa A, Carter VM, et al. Evaluation of community perceptions and prevention practices related to Ebola virus as part of outbreak preparedness in Uganda, 2020. *Glob Health Sci Pract*. 2022;10(3):e2100661. https://doi.org/10.9745/GHSP-D-21-00661

	All districts N = 3,485 % (95%CI)	Low-risk districts		High-risk districts			
		Busia N=634 n (%)	Lamwo N=593 n (%)	Arua N=506 n (%)	Kampala N=511 n (%)	Kisoro N=612 n (%)	Kasese N=629 n (%)
Would buy fresh vegetables from survivor shopkeeper ¥	47.3 (43.8, 50.9)	40.8 (34.0,48.0)	67.0 (62.0,71.6)	58.5 (50.6,65.9)	47.1 (41.2,53.1)	18.6 (13.1,25.6)	48.8 (39.2,58.6)
**Attitudes toward safe burial practices**							
Accept safe alternatives to traditional burial rituals	80.6 (77.1, 83.6)	84.5 (78.9,88.8)	78.1 (69.6,84.8)	81.7 (59.6,93.1)	76.8 (71.8,81.2)	83.5 (77.0,83.4)	86.1 (79.4,90.8)
**Intention if family member is suspected of EVD ¥**							
Take the family member to a health facility	52.4 (46.3, 58.5)	67.3 (59.5,74.2)	59.4 (48.5,69.4)	46.5 (32.2,61.4)	51.5 (40.3,62.5)	42.6 (37.0,48.4)	51.4 (45.2,57.6)
Report to district health authorities	43.3 (38.8, 48.0)	29.2 (24.2,34.9)	57.5 (51.7,63.1)	48.9 (40.6,57.2)	42.8 (34.4,51.5)	60.9 (53.7,67.7)	37.2 (31.5,43.3)
Avoid all physical contact	10.3 (7.8, 13.6)	4.0 (2.4,6.6)	15.4 (10.6,21.9)	13.4 (9.8,18.2)	8.4 (4.2,16.0)	11.6 (8.7,15.4)	16.5 (13.8,19.6)
Do nothing	5.2 (3.8, 6.9)	12.2 (8.3,17.6)	4.8 (2.5,8.8)	10.0 (3.7,24.0)	2.5 (1.7,3.8)	5.6 (3.4,9.0)	5.1 (2.1,11.9)
Help care for the family member at home	2.6 (1.7,4.0)	2.9 (1.2,6.9)	9.2 (6.3,13.2)	3.4 (1.9,6.1)	1.7 (0.5,5.6)	0.7 (0.3,1.6)	3.3 (1.8,5.9)
Check person’s temperature	1.5 (0.7,3.2)	0.5 (0.1,2.1)	1.5 (0.6,3.4)	0.2 (<0.1,1.4)	2.1 (0.8,5.4)	0.5 (0.1,2.0)	2.0 (0.3,12.3)
Hide the EVD suspected family member	<0.1 (<0.1, 0.1)	0	0.4 (0.1,1.4)	0	0	0	0.1 (<0.1,0.7)
**Self-reported participation in recent funeral or burial**							
Self-reported participation in a funeral/burial in past month	39.3 (35.7, 43.0)	47.6 (40.4,54.8)	29.8 (21.6,39.7)	58.8 (45.3,71.2)	24.3 (19.0,30.4)	49.0 (35.8,62.3)	67.9 (60.8,74.1)
Religious leader prayed for the deceased ♀	84.9 (78.7, 89.5)	91.9 (87.1,95.0)	88.1 (80.4,93.0)	90.2 (82.0,94.9)	79.9 (67.4,88.4)	86.9 (78.8,92.3)	90.3 (85.7,93.5)
Family members observed burial from a distance ♀	6.2 (3.8, 9.9)	13.8 (7.8,23.3)	3.1 (1.5,6.4)	2.6 (1.1,6.4)	5.5 (2.0,13.8)	0.8 (0.2,2.8)	9.8 (4.9,18.7)
Attendees performed rituals involving contact with corpse ♀	25.5 (17.4, 35.8)	18.5 (11.4,28.5)	18.7 (11.3,29.2)	20.1 (14.8,26.6)	26.4 (12.3,47.9)	13.5 (9.4,19.2)	40.2 (34.9,45.7)
Attendees touched each other (e.g. hug, handshakes) ♀	50.8 (42.7, 58.8)	66.2 (56.9,74.4)	52.1 (41.9,62.2)	27.4 (19.4,37.1)	51.6 (36.3,66.6)	44.7 (37.9,51.6)	58.8 (48.3,68.6)
Respondent had physical contact with the corpse ♀	11.1 (8.4, 14.7)	8.1 (4.7,13.7)	13.7 (9.5,19.5)	19.3 (12.8,28.0)	7.7 (3.7,15.1)	6.9 (3.8,12.3)	19.2 (12.4,28.4)
**Self-reported EVD prevention practices †**							
Self-reported one or more Ebola prevention practice	67.3 (63.3, 71.1)	73.0 (61.4,82.2)	71.3 (61.5,79.3)	84.6 (76.3,90.3)	57.4 (50.3,64.2)	67.7 (53.8,79.0)	80.8 (77.0,84.0)
Wash hands with soap and water more frequently	57.1 (52.0, 62.0)	59.0 (42.9,73.4)	53.8 (42.6,64.8)	68.4 (54.7,79.5)	50.2 (41.8,58.6)	59.2 (42.7,73.9)	69.8 (64.1,74.7)
Avoid physical contact with suspected Ebola patients	22.9 (19.3, 26.8)	21.6 (13.0,33.8)	35.9 (27.7,45.0)	25.9 (20.2,32.5)	20.6 (14.9,27.7)	22.9 (18.0,28.5)	23.8 (16.2,33.5)
Avoid burials that involve contact with a corpse	13.3 (10.1, 17.2)	15.1 (9.7,22.7)	8.1 (4.1,15.3)	20.9 (15.0,28.3)	13.1 (7.8,21.3)	6.4 (4.0,10.0)	11.1 (8.1,15.1)
Reported all three prevention practices	4.0 (2.0, 7.8)	3.2 (1.1,8.9)	1.4 (0.5,4.0)	5.7 (2.8,11.1)	4.5 (1.4,13.7)	1.9 (0.8,4.6)	3.5 (1.4,8.2)
Other responses:							
Wash my hands with just water more often	63.0 (57.6,68.0)	62.1 (45.3,76.5)	59.6 (46.4,71.6)	69.3 (55.5,80.4)	53.2 (44.0,62.1)	82.5 (75.4,87.9)	84.3 (80.8,87.2)

Supplement to: Musaazi J, Namageyo-Funa A, Carter VM, et al. Evaluation of community perceptions and prevention practices related to Ebola virus as part of outbreak preparedness in Uganda, 2020. *Glob Health Sci Pract*. 2022;10(3):e2100661. https://doi.org/10.9745/GHSP-D-21-00661

	**All districts N = 3,485** % (95%CI)	**Low-risk districts**		**High-risk districts**			
		Busia N=634 n (%)	Lamwo N=593 n (%)	Arua N=506 n (%)	Kampala N=511 n (%)	Kisoro N=612 n (%)	Kasese N=629 n (%)
Clean my hands with other disinfectants more often	15.5 (12.2,19.5)	21.7 (14.9,30.4)	5.6 (2.4,12.3)	9.0 (3.3,22.2)	22.1 (16.1,29.6)	1.5 (0.6,4.0)	4.0 (2.6,6.0)
Try to avoid crowded places	14.0 (9.5,20.3)	7.1 (3.6,13.4)	19.6 (12.6,29.0)	14.6 (9.2,22.4)	11.7 (4.6,26.6)	9.4 (6.5,13.6)	26.5 (22.3,31.1)
Drink a lot of water / juice	2.0 (1.0,3.7)	1.7 (0.6,4.7)	0	4.6 (1.4,14.0)	1.7 (0.5,5.7)	2.8 (1.3,6.1)	1.2 (0.4,3.6)
Drink traditional herbs	0.4 (0.1,1.2)	0.7 (0.1,5.6)	0	0.4 (0.1,3.1)	0.3 (<0.1,2.8)	0.4 (0.1,1.5)	0.4 (0.1,2.9)
Take antibiotics (e.g. penicillin, amoxilin)	0.2 (<0.1,0.6)	0	0.7 (0.1,4.8)	0.2 (<0.1,1.6)	0	0	0.7 (0.1,4.0)
Wear gloves	0.3 (0.1,0.9)	0	0	0.4 (0.1,3.3)	0.3 (<0.1,2.3)	0	0.6 (0.2,1.7)
Wash with salt and hot water	0.6 (0.2,1.4)	0.8 (0.1,5.6)	0.4 (0.1,2.6)	1.4 (0.3,6.4)	0.3 (<0.1,2.8)	0	1.0 (0.3,3.0)

¶ Design based F-statistic P values; comparing High risk versus Low risk districts.

¥ Missing values: Knowledge of EVD prevention and treatment (n=66, 2%), Knowledge of EVD signs \symptoms (n=792, 23%), Traditional healers can successfully treat EVD (n=66, 2%), Spiritual healers can successfully treat EVD (n=66, 2%), EVD is transmissible by air (n=518, 15%), Comprehensive knowledge (n= 523, 15%). Would welcome back EVD survivor into the community (n=79, 2%), Would buy fresh vegetables from EVD survivor shopkeeper (n=91, 3%), Ebola survivor student does not put class at risk of EVD (n=92, 3%), Would avoid touching or washing the corpse of a family member (n=48, 1%), Intended practice if family member is suspected of EVD (n=627, 18%), Participated in a funeral/burial in past month (n=30, <1%), Features of the funeral/burial ceremony (n=64, 4%), Respondents had physical contact with corpse at the funeral/burial (n=33, 2%)

† Denominator is number of respondents who reported that they took action to avoid getting EVD.

♀ Denominator is number of respondents who self-reported to have participated in a funeral/burial in past month

s
